# Supplementary material for: Within-species floral evolution reveals convergence in adaptive walks during incipient pollinator shift
Source: Nat Commun. 2025 Mar 19;16:2721. doi: 10.1038/s41467-025-57639-3 (PMC11923230; doi:10.1038/s41467-025-57639-3)
Supplement: Supplementary file 1 — Supplementary Information [file 41467_2025_57639_MOESM1_ESM.pdf]

SUPPLEMENTARY INFORMATION

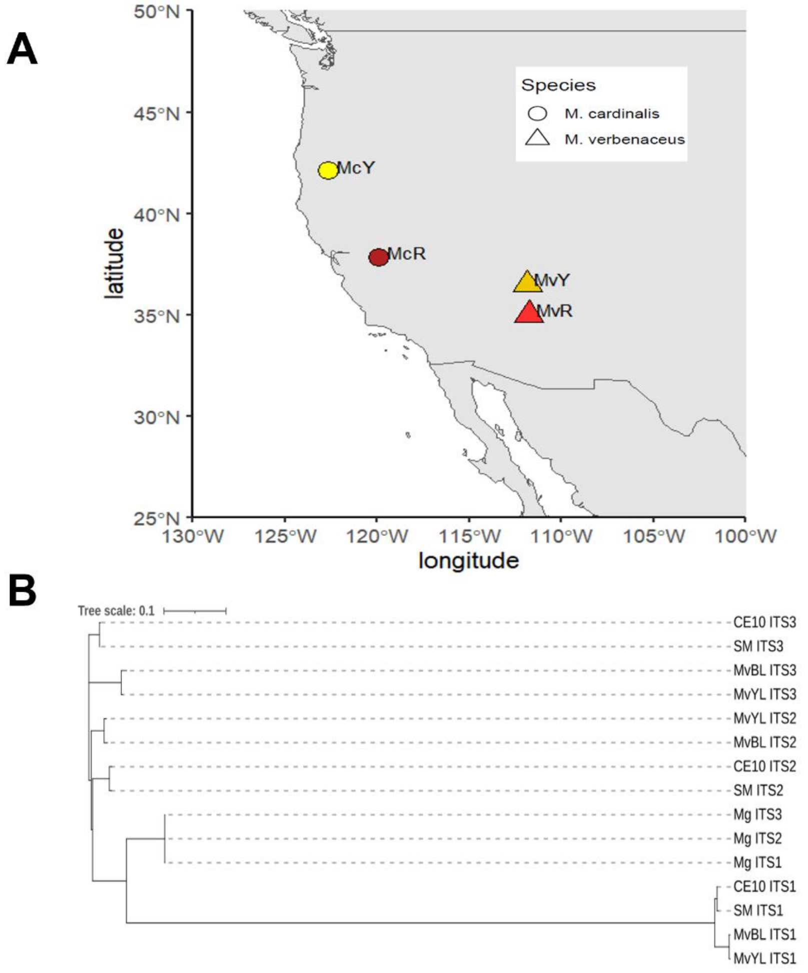

Figure S1. Yellow morphs arose independently from red conspecific forms in *Mimulus verbenaceus* and *M. cardinalis*. (A) Map of approximate collection locations of seed of focal lines, showing geographically distant populations of novel yellow morphs, consistent with their repeated independent origins. Shape of point shows species as denoted in key; color denotes red (McR, MvR) vs yellow (McY, MvY) morphs. Map was plotted in R v4.2.2 using packages sf v 1.0-17 (Pebesma, 2018; Pebesma and Bivand, 2023), rnaturalearth v1.0.1 (Massicotte and South, 2023), and rnaturalearthdata v1.0.0 (South and Massicotte, 2024) with a free, public domain map dataset from Natural Earth (https://www.naturalearthdata.com/). **(B)** Evolutionary relationships of focal lines of *M. verbenaceus* (MvBL/MvY and MvYL/MvR) and *M. cardinalis* (CE-10/McR and SM/McY), with congener *M. guttatus* (Mg) as outgroup; the evolutionary history was inferred by using the Maximum Likelihood method and Tamura-Nei model on 3 ITS sequences from the genomes of the four focal morphs and outgroup *Mimulus guttatus* (Mg). See Supplemental Data 12 for sequences. Analysis confirms that yellow morphs (MvYL/MvY and SM/McY) are most closely related to their red conspecific lines (MvBL/MvR and CE-10/McR, respectively), again consistent with their repeated independent origins. Abbreviations: MvYL = MvY, MvBL = MvR, SM = McR, CE-10 = McR.

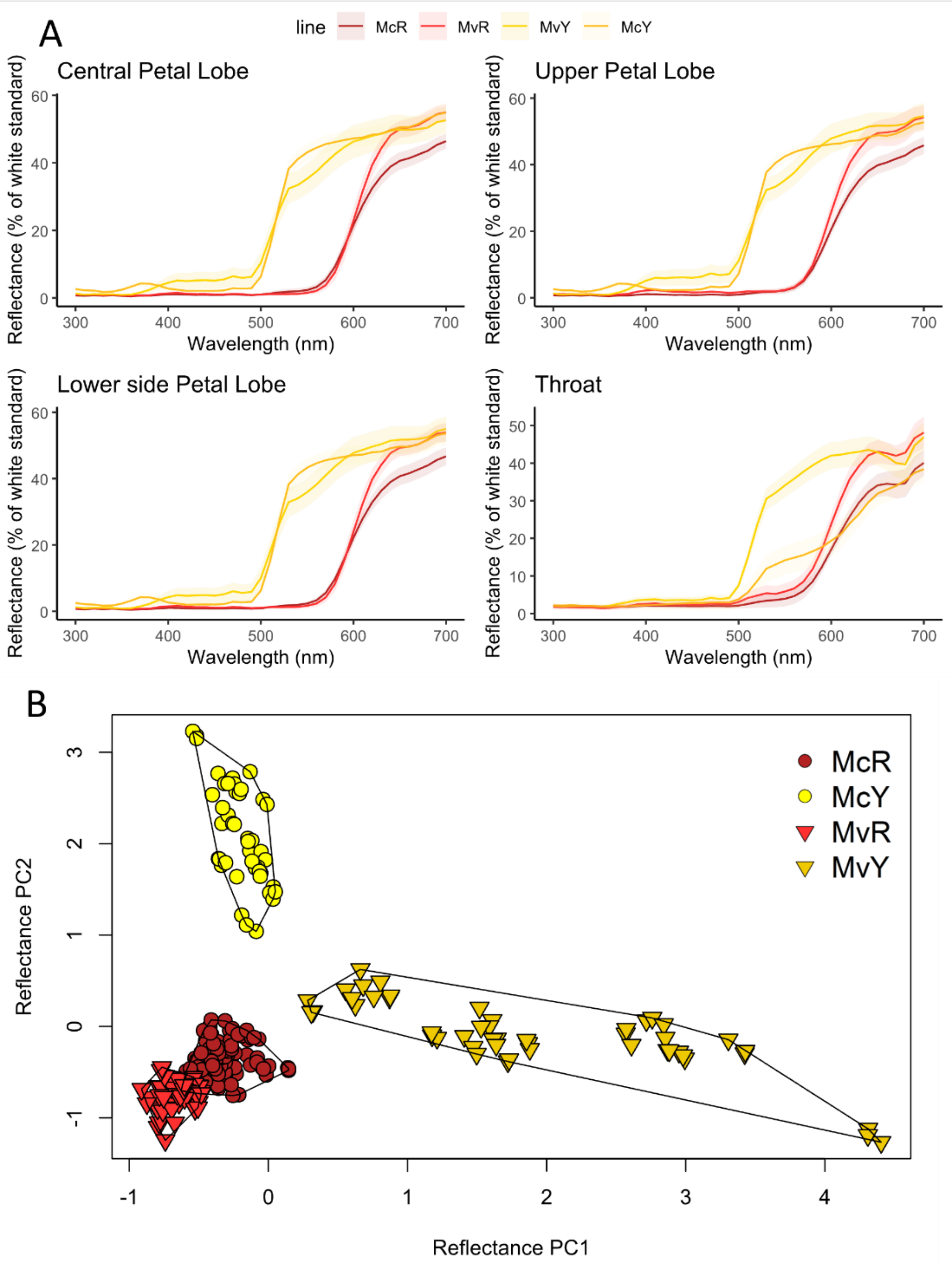

Figure S2. Similar reflectance patterns across corolla tissues and among red morphs; yellow morphs differ in color. (A) Reflectance curves of all four corolla tissues measured. Central line and shading shows mean reflectance +/- standard deviation. Central petal lobe: N= 94 flowers; Upper petal lobe: N = 96 flowers; Lower side petal lobe: N = 95 flowers; Throat: N = 59 flowers. (B) Plot of PC1 and PC2 of PCA from reflectance data of central petal lobes of all plant lines. For the purposes of visualization, all technical replicates are plotted on the PCA (N = 283 total reflectance measurements taken from 94 flowers). Detailed sample sizes per line are given in Table S1. Source data are provided as a Source Data file.

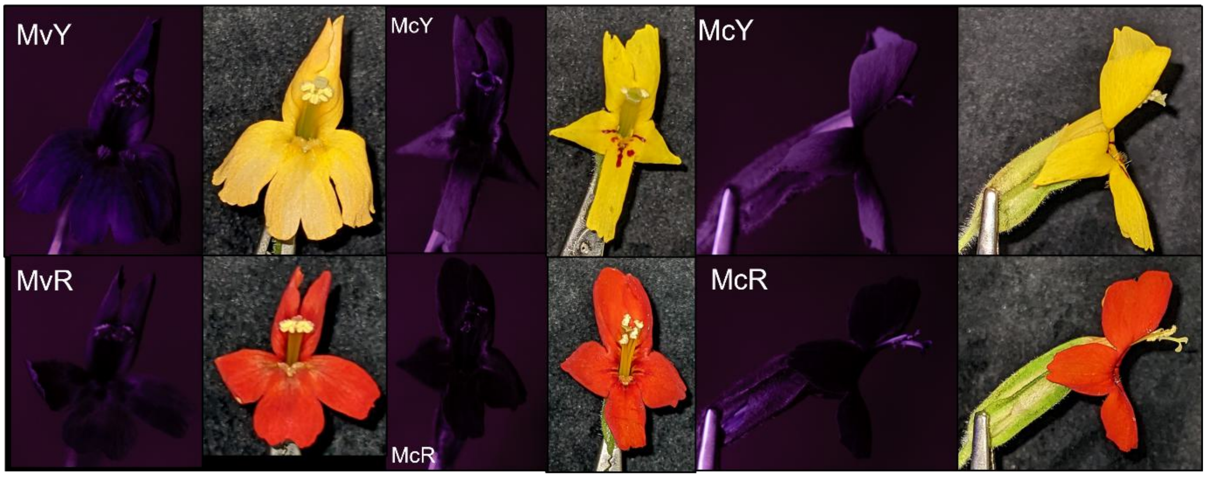

Figure S3. Yellow morphs reflect somewhat in the UV spectrum; red morphs not at all. UV photography alongside visual spectrum photography of the same flowers from each plant line. Left two panes: *M. verbenaceus* (MvY, left top row; MvR, left bottom row). Lines of *M. cardinalis* (McY, center and right top row; McR, center and right bottom row) are also shown in side-view given their highly reflexed petal lobes.

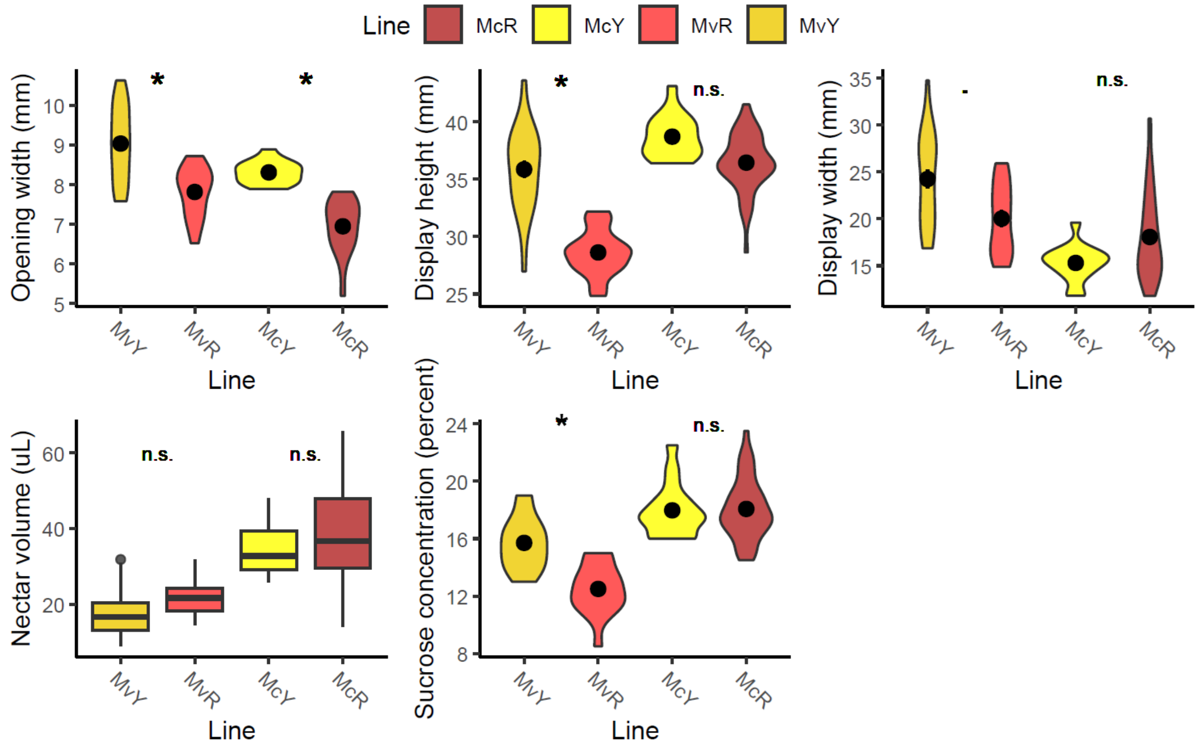

Figure S4. Floral morphology and nectar variation among floral color morphs. Violin plots of additional floral morphology and nectar traits by plant line. Dot shows mean and bars +/- standard error. For nectar volume, boxplot shows median value (center bars), first and third quartiles (upper and lower hinges), points within 1.5*interquartile range of hinges (whiskers), and outlying points (large points). Asterisks denote significant pairwise differences between morphs within species based on 95% confidence intervals of least squares means with Tukey adjustments for multiple comparisons ($p < 0.05$); points (.) denote marginal differences ($0.1 > p > 0.05$); "n.s" denotes no significant difference ($p > 0.1$) based on same tests as above. Opening width: N= 101 flowers (MvR-MvY: p= 0.0002; McR-McY: p< 0.0001). Display height: N= 102 flowers (MvR-MvY: p <.0001; McR-McY: p= 0.09). Display width: N= 102 flowers (MvR-MvY: p= 0.054; McR-McY: p= 0.22). Nectar volume (uL): N = 96 flowers (MvR-MvY: p= 0.8; McR-McY: p=0.6). Sucrose concentration (percent sucrose): N = 96 flowers (MvR-MvY: p= 0.0017; McR-McY: p= 0.99). Detailed sample sizes per line and statistics are given in Tables S1, S3. Source data are provided as a Source Data file.

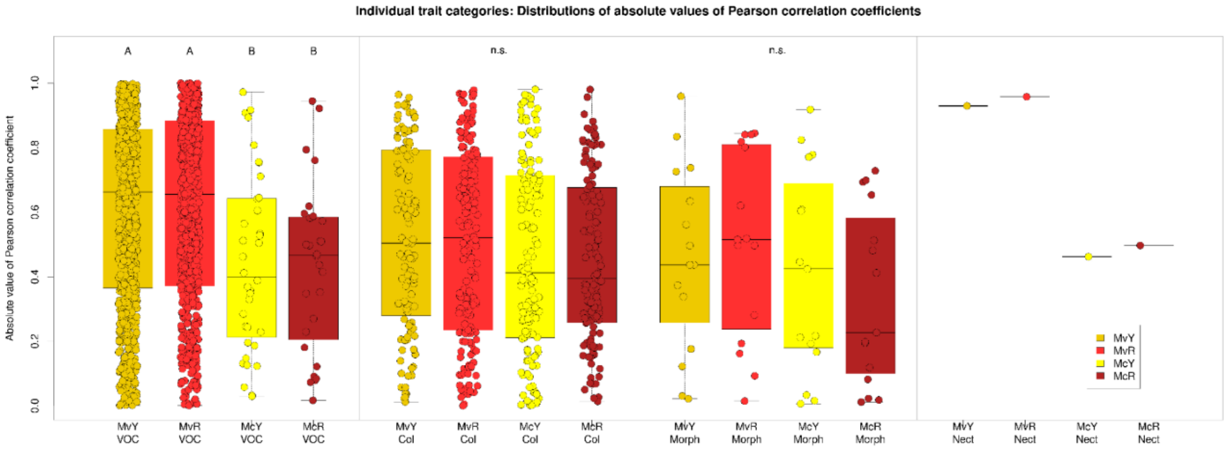

Figure S5. Distributions of absolute Pearson Correlation Coefficient values within trait categories. Traits (scent, color, morphology, nectar) are sorted by trait type. VOC: volatile organic compounds; Col: pigment and reflectance spectrophotometry (latter values from principal component analysis); Morph: morphological traits; Nect: nectar traits. Statistical analysis of Nec was not performed given only one correlation value was available per line. Letters above or below boxes indicate statistically significant differences. n.s., not significant. Statistics: VOCs: one-way ANOVA, df = 3, F = 7.330, p = 7.065e-5; Col: one-way ANOVA, df = 3, F = 1.727, p = 0.160; Morph: one-way ANOVA, df = 3, F = 0.866, p = 0.464. Data from 5 individual plants. Box plot definition: box height = interquartile range; line = median; whiskers = closest data point to 1.5 times interquartile range. Source data are provided as a Source Data file.

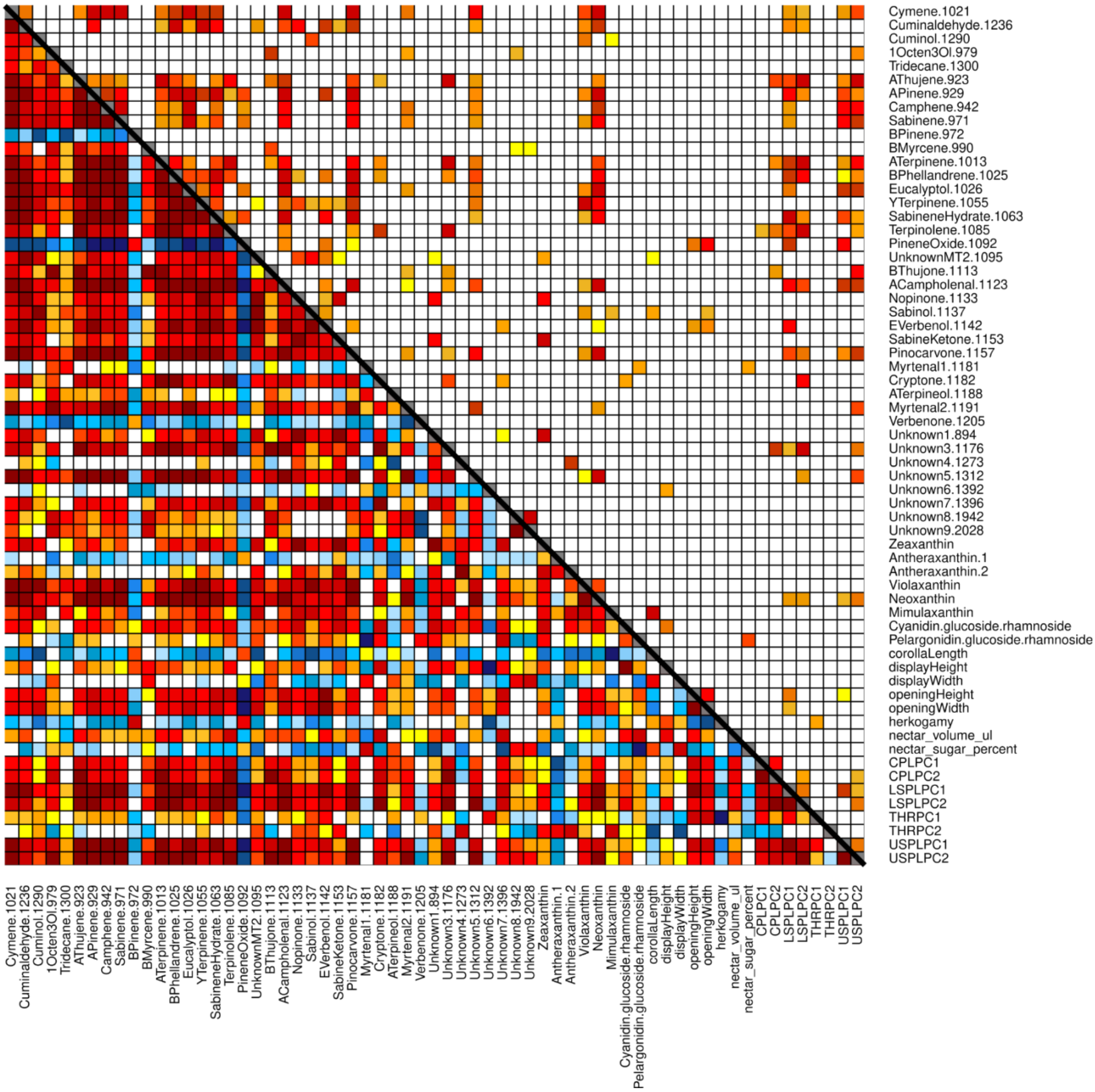

Figure S6. All-trait correlations in MvY. The lower left triangle displays Pearson correlation coefficients (blue = negatively correlated, yellow to red = positively correlated, darker colors are more strongly correlated) and the upper right triangle displays the p-values of correlations (white is $p > 0.05$, darker red is more significant). CPLPC1-2, central petal lobe reflectance PC1-2; LSPLPC1-2, lower side petal lobe reflectance PC1-2; THRPC1-2, corolla throat reflectance PC1-2; USPLPC1-2, upper side petal lobe reflectance PC1-2. Data from 5 individual plants. Source data are provided as a Source Data file.

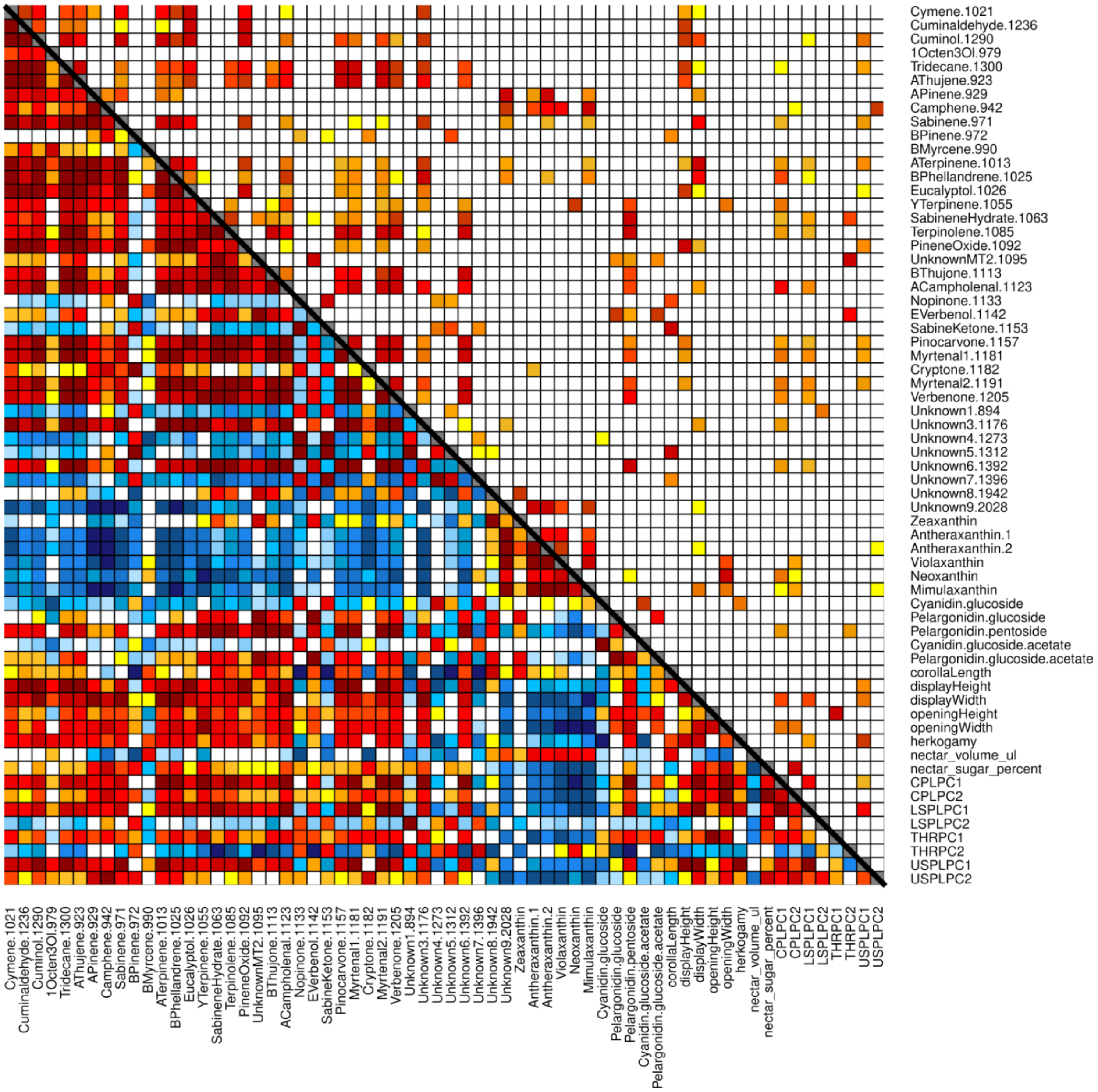

Figure S7. All-trait correlations in MvR. The lower left triangle displays Pearson correlation coefficients (blue = negatively correlated, yellow to red = positively correlated, darker colors are more strongly correlated) and the upper right triangle displays the p-values of correlations (white is $p > 0.05$, darker red is more significant). CPLPC1-2, central petal lobe reflectance PC1-2; LSPLPC1-2, lower side petal lobe reflectance PC1-2; THRPC1-2, corolla throat reflectance PC1-2; USPLPC1-2, upper side petal lobe reflectance PC1-2. Data from 5 individual plants. Source data are provided as a Source Data file.

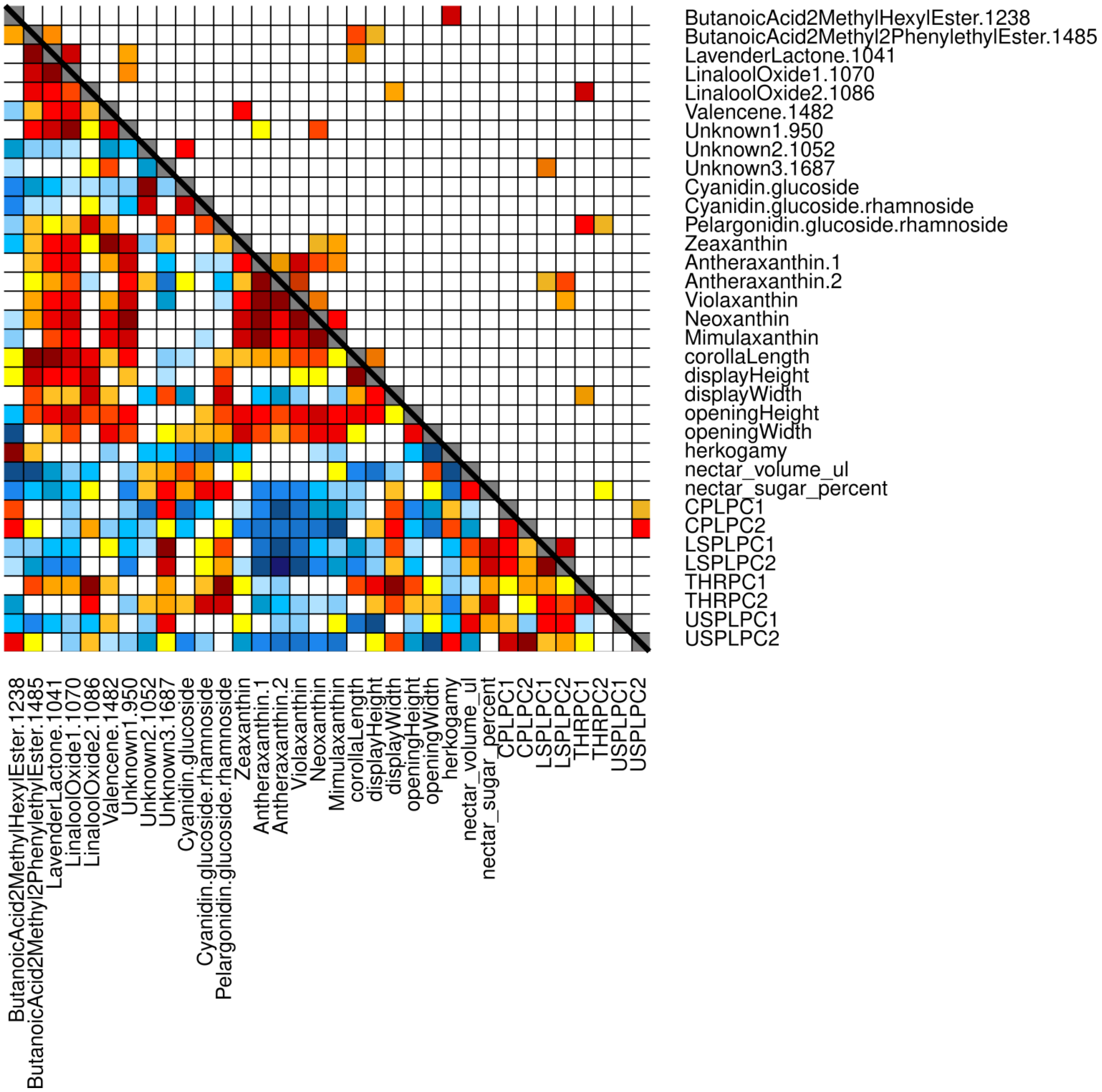

Figure S8. All-trait correlations in McY. The lower left triangle displays Pearson correlation coefficients (blue = negatively correlated, yellow to red = positively correlated, darker colors are more strongly correlated) and the upper right triangle displays the p-values of correlations (white is p > 0.05, darker red is more significant). CPLPC1-2, central petal lobe reflectance PC1-2; LSPLPC1-2, lower side petal lobe reflectance PC1-2; THRPC1-2, corolla throat reflectance PC1-2; USPLPC1-2, upper side petal lobe reflectance PC1-2. Data from 5 individual plants. Source data are provided as a Source Data file.

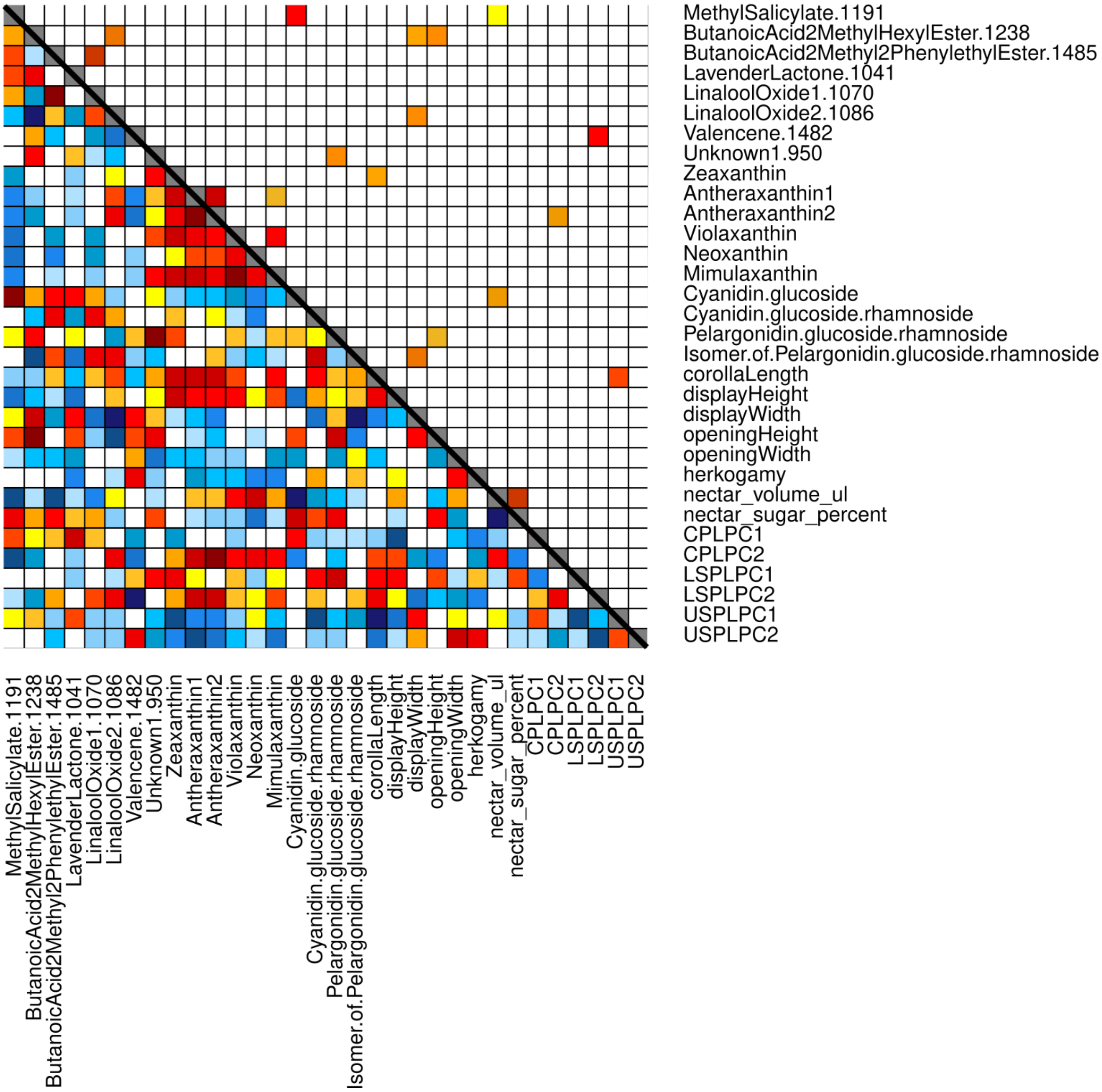

Figure S9. All-trait correlations in McR. The lower left triangle displays Pearson correlation coefficients (blue = negatively correlated, yellow to red = positively correlated, darker colors are more strongly correlated) and the upper right triangle displays the p-values of correlations (white is $p > 0.05$, darker red is more significant). CPLPC1-2, central petal lobe reflectance PC1-2; LSPLPC1-2, lower side petal lobe reflectance PC1-2; USPLPC1-2, upper side petal lobe reflectance PC1-2. Data from 5 individual plants. Source data are provided as a Source Data file.

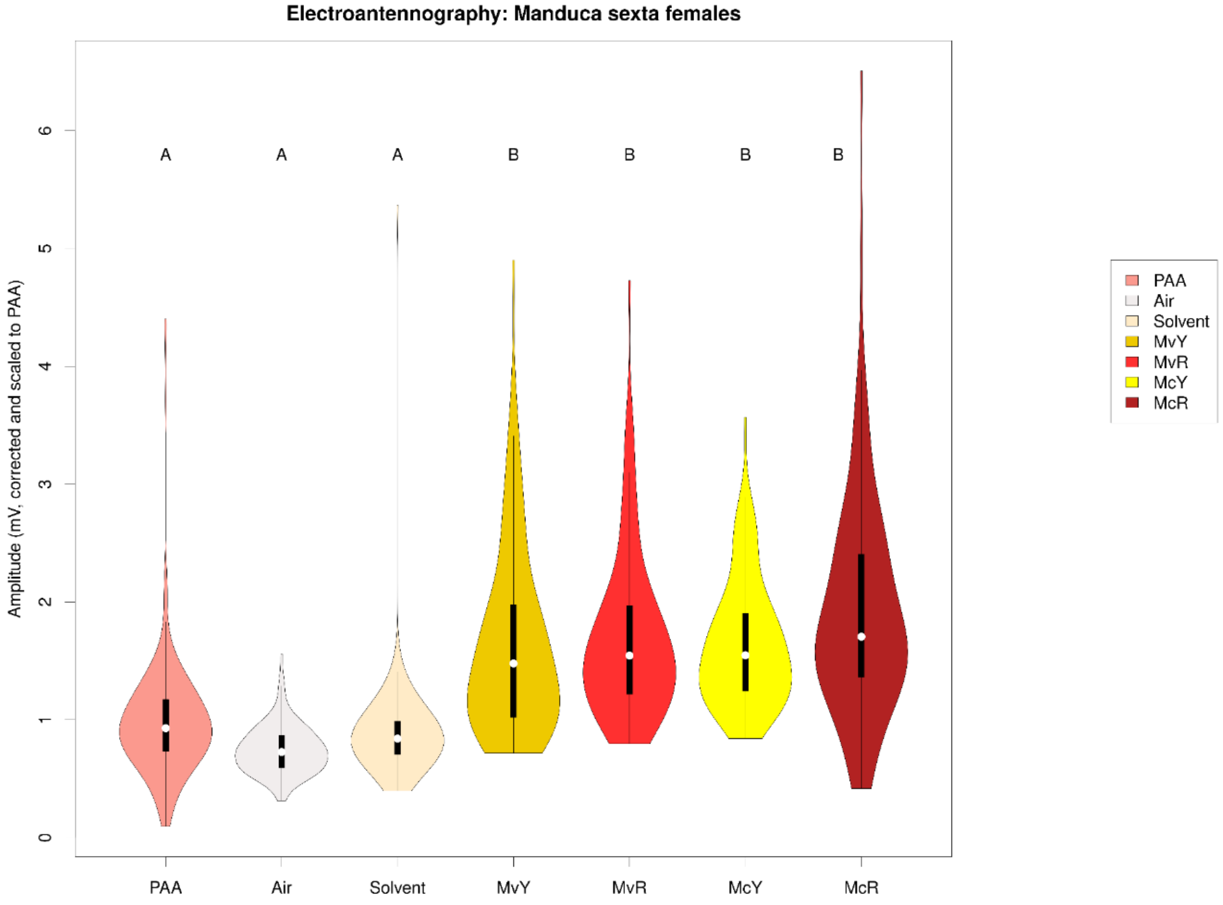

Figure S10: EAG responses to scent stimuli by seven female *Manduca sexta* hawkmoths. Stimuli include phenylacetaldehyde (PAA, a floral VOC known to be detectable by *Manduca sexta*), air (negative control), extraction solvent alone, and floral scent extractions of MVYL, MVBL, SM, and CE10. Letters above violins indicate statistically significant differences. Statistics: linear mixed model (fixed effect: stimulus; random effects: trial and stimulus nested within trial), followed by Type II Wald chi-square test (df = 6, $X^2 = 95.085$, $p < 2.2e-16$). N = 7 moths; PAA: 189 presentations across all moths; air: 105; solvent: 105; MvY: 105; MvR: 105: McY: 102; McR: 108. Source data are provided as a Source Data file.

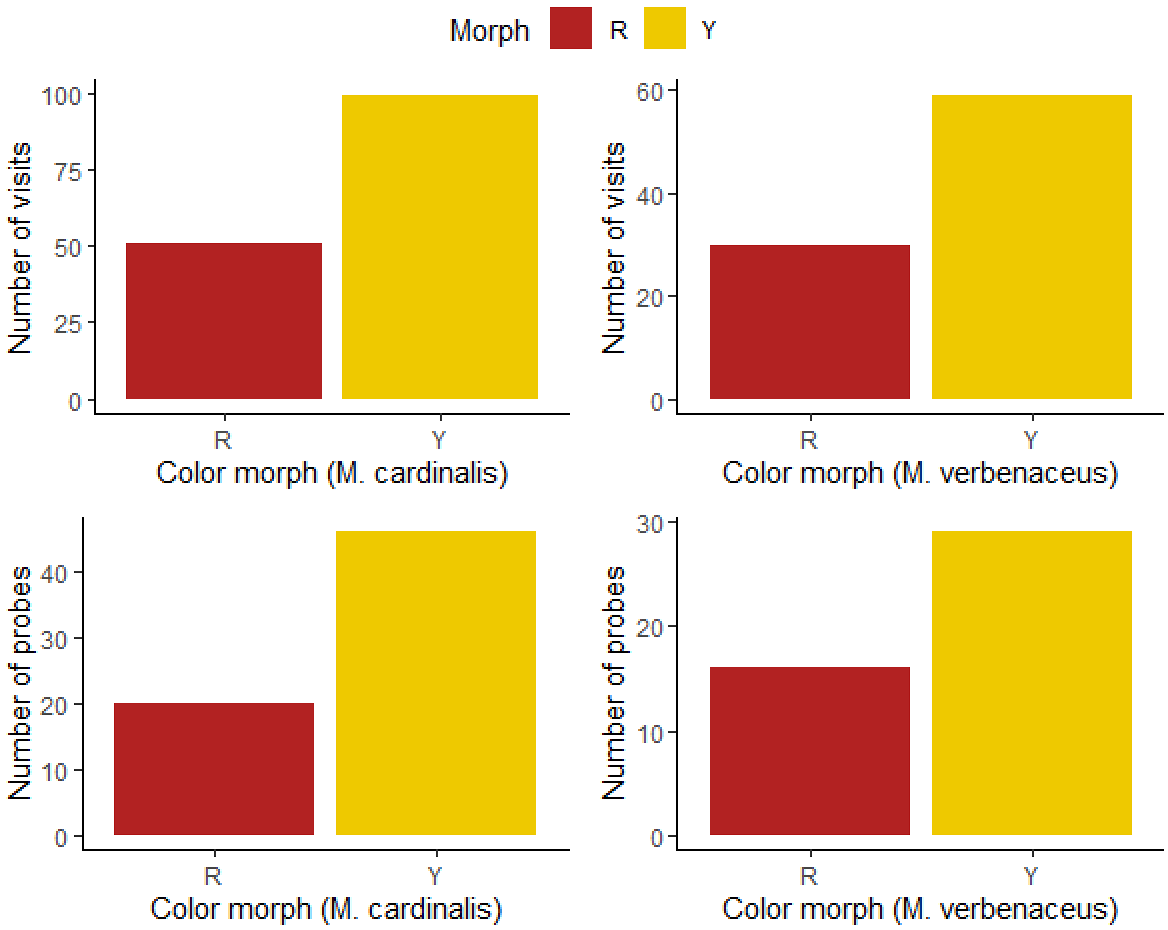

Figure S11. Bees show a consistent 2:1 preference overall for yellow morphs over red. Total number of floral visits by bumblebees to each floral color morph for each within-species pairwise comparison, pooled across all trials (a different naive bumblebee was used in each trial); N=10 trials per plant species comparison. Color morphs: M. cardinalis: R = McR, Y= McY; M. verbenaceus: R= MvR, Y= MvY. Source data are provided as a Source Data file.

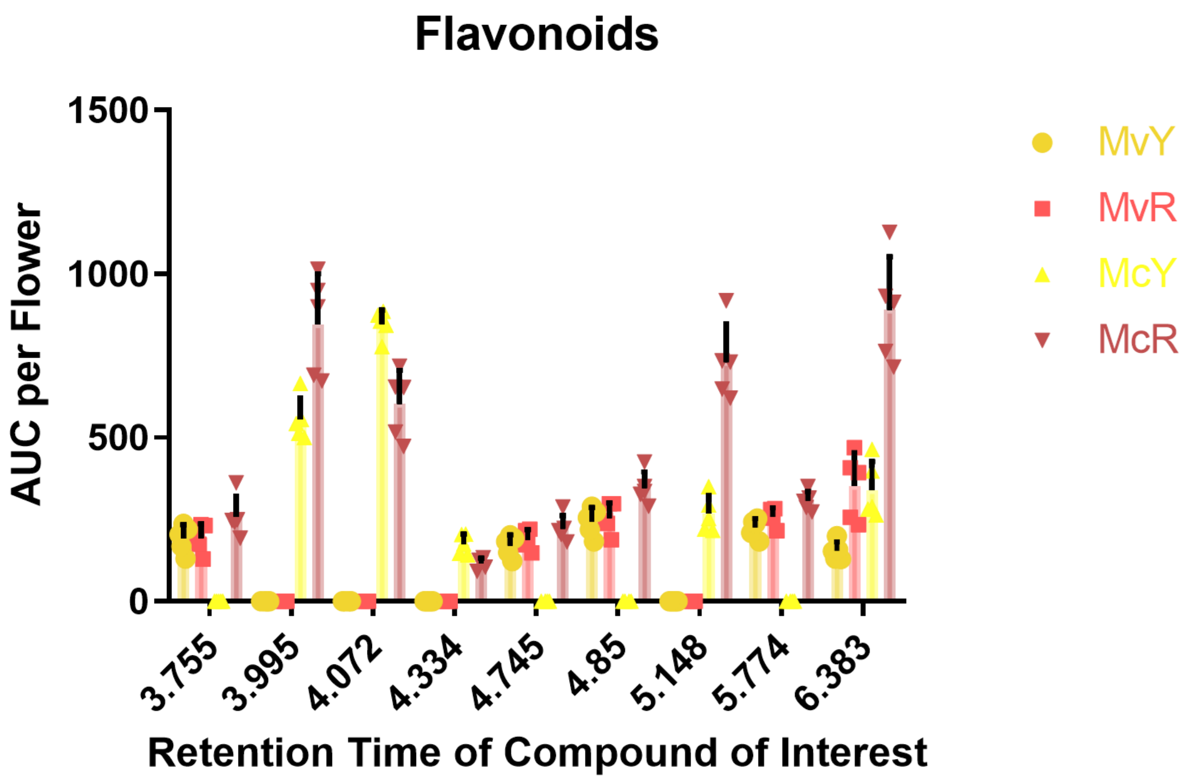

Figure S12. Flavonoid accumulation as peaks detected at wavelength 350nm. As individual compounds were unable to be identified, retention times using the LCMS method described in the main text denote the identifier for the compounds being compared. A two-way ANOVA of compounds can be found in Supplementary Table 8. (n=5) Data are presented as mean values +/- SEM. Source data are provided as a Source Data file.

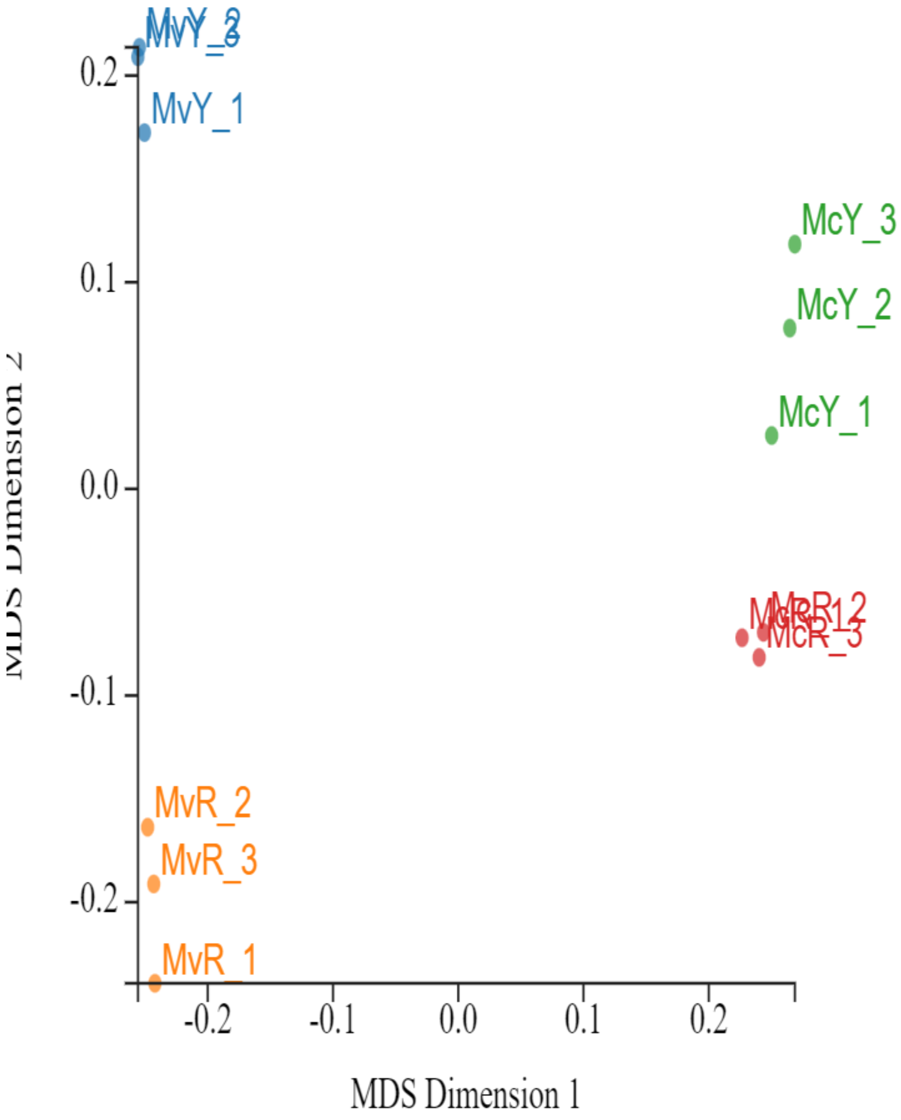

Figure S13. Lines cluster together based on whole transcriptome data. NMDS of whole transcriptomes of the four focal morphs (plotted as different colors) visualized in Degust. N= 3 plants per line. Source data are provided as a Source Data file.

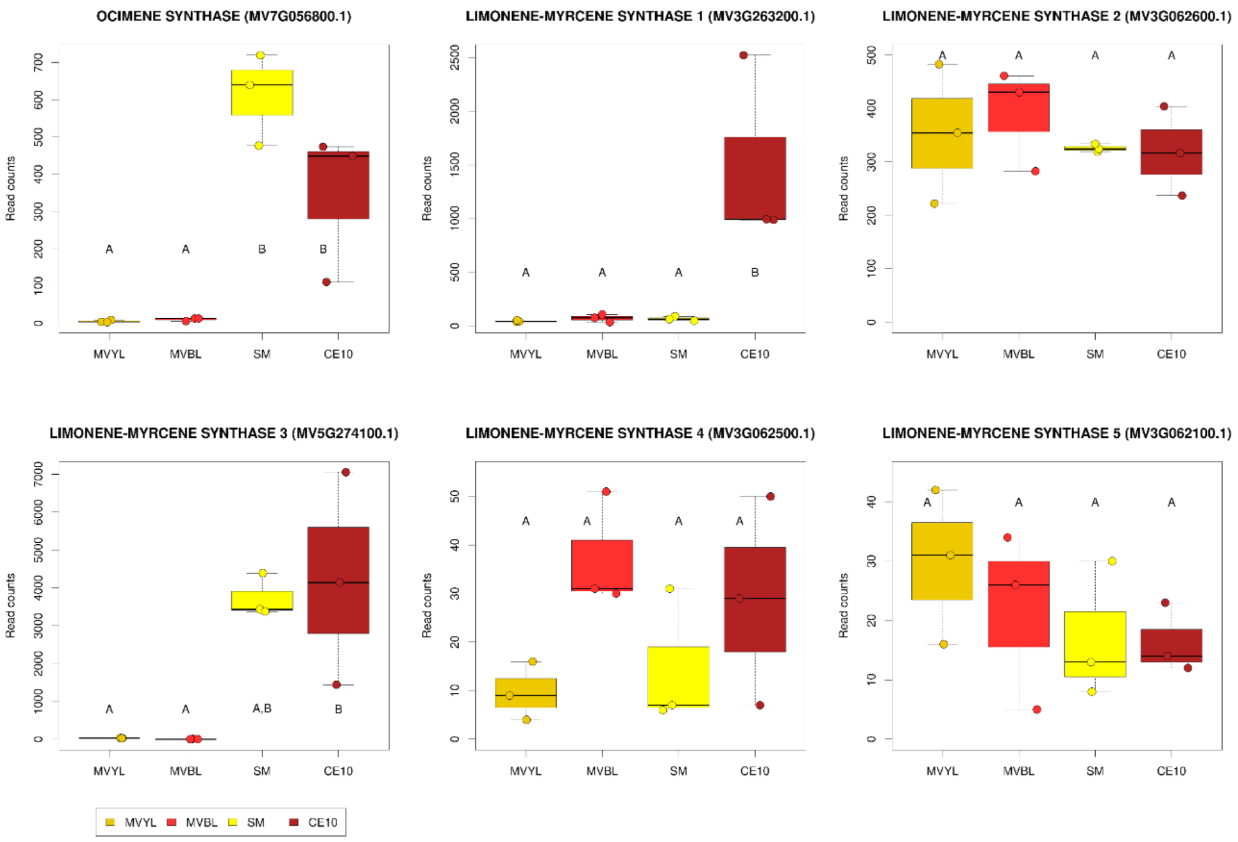

Figure S14. Gene expression data (mapped read counts) from six floral scent genes previously identified in *Mimulus* section *Erythranthe*. *Mimulus verbenaceus* MVBL gene ID listed above each plot. Letters above or below boxes indicate statistically significant differences. N = 3 biological replicates for each line. Abbreviations: MVYL = MvY, MVBL = MvR, SM = McR, CE10 = McR. Statistics: one-way ANOVA (*OCIMENE SYNTHASE*: df = 3, F = 18.219, p = 0.0006; *LIMONENE-MYRCENE SYNTHASE 1*: df = 3, F = 7.937, p = 0.009; *LIMONENE-MYRCENE SYNTHASE 2*: df = 3, F = 0.389, p = 00.764; *LIMONENE-MYRCENE SYNTHASE 3*: df = 3, F = 7.683, p = 0.010; *LIMONENE-MYRCENE SYNTHASE 4*: df = 3, F = 2.307, p = 0.153; *LIMONENE-MYRCENE SYNTHASE 5*: df = 3, F = 0.806, p = 0.525). Box plot definition: box height = interquartile range; line = median; whiskers = closest data point to 1.5 times interquartile range. Source data are provided as a Source Data file.

SUPPLEMENTAL TABLES

TABLE S1. Sample sizes of biological replicates (flowers and individual plants) used for each set of experiments. Bold indicates headings/ categories.

**N (# flowers)**						**N (# individuals)**				
**Dataset**	**McR**	**McY**	**MvR**	**MvY**		**Dataset**	**McR**	**McY**	**MvR**	**MvY**
**corollaLength**	61	15	18	26		**corollaLength**	20	5	6	6
**displayHeight**	46	15	18	23		**displayHeight**	15	5	6	6
**displayWidth**	46	15	18	23		**displayWidth**	15	5	6	6
**openingHeight**	46	15	17	23		**openingHeight**	15	5	6	6
**openingWidth**	46	15	17	23		**openingWidth**	15	5	6	6
**pistilLength**	15	15	18	18		**pistilLength**	5	5	6	6
**stamenLength**	15	15	18	18		**stamenLength**	5	5	6	6
**nectar (volume, % sugar)**	44	17	18	17		**nectar (volume, % sugar)**	14	5	6	5
**reflectance_CentrePetalLobe**	45	15	18	16		**reflectance_CentrePetalLobe**	16	5	6	5
**reflectance_LowerSidePetalLobe**	46	15	18	16		**reflectance_LowerSidePetalLobe**	16	5	6	5
**reflectance_Throat**	10	15	18	16		**reflectance_Throat**	8	5	6	5
**reflectance_UpperPetalLobe**	47	15	18	16		**reflectance_UpperPetalLobe**	16	5	6	5
**anthocyanins**	5	5	5	5		**anthocyanins**	5	5	5	5
**carotenoids**	5	5	5	5		**carotenoids**	5	5	5	5
**scentCollections (2 flowers/sample)**	36	15	18	17		**scentCollections (2 flowers/sample)**	15	5	6	6
**WGS**	NA	NA (single leaf)	NA	NA (single leaf)		**WGS**	0	1	0	1
**RNAseq**	3	3	3	3		**RNAseq**	3	3	3	3

TABLE S2. Total anthocyanins and total carotenoids vary among lines. Pairwise comparisons of absorbance for total anthocyanins (at 525nm) and total carotenoids (at 450nm) by line nested within species. Pairwise comparisons following ANOVA (see main text for details) based on 95% confidence interval of least squares means (lsmeans() function) following the Tukey method for p-value adjustment for multiple comparisons. N= 20 flowers (5 per line). Bold indicates headings/ categories.

Anthocyanins

Least squares means (lsmeans())

line	sp	lsmean	SE	df	lower.CL	upper.CL
McR	Mcardinalis	3.939	0.196	16	3.524	4.355
McY	Mcardinalis	0.641	0.196	16	0.225	1.056
MvR	Mverbenaceus	4.766	0.196	16	4.351	5.181
MvY	Mverbenaceus	0.184	0.196	16	-0.232	0.599

Pairwise contrasts between line nested within species

contrast	estimate	SE	df	t.ratio	p
McR Mcardinalis - McY Mcardinalis	3.299	0.277	16	11.91	<.0001
McR Mcardinalis - MvR Mverbenaceus	-0.827	0.277	16	-2.985	0.0393
McR Mcardinalis - MvY Mverbenaceus	3.756	0.277	16	13.56	<.0001
McY Mcardinalis - MvR Mverbenaceus	-4.126	0.277	16	-14.895	<.0001
McY Mcardinalis - MvY Mverbenaceus	0.457	0.277	16	1.65	0.3806
MvR Mverbenaceus - MvY Mverbenaceus	4.583	0.277	16	16.545	<.0001

Confidence level used: 0.95
P-value adjustment based on Tukey method for a family of four estimates.

Carotenoids

Least squares means (lsmeans())

line	sp	lsmean	SE	df	lower.CL	upper.CL
McR	Mcardinalis	5.37	0.433	16	4.45	6.29
McY	Mcardinalis	7.26	0.433	16	6.35	8.18
MvR	Mverbenaceus	3.31	0.433	16	2.39	4.23
MvY	Mverbenaceus	5.95	0.433	16	5.03	6.86

Pairwise contrasts between line nested within species

contrast	estimate	SE	df	t.ratio	p
McR Mcardinalis - McY Mcardinalis	-1.894	0.613	16	-3.09	0.032
McR Mcardinalis - MvR Mverbenaceus	2.063	0.613	16	3.366	0.0185
McR Mcardinalis - MvY Mverbenaceus	-0.575	0.613	16	-0.938	0.785
McY Mcardinalis - MvR Mverbenaceus	3.957	0.613	16	6.456	<.0001
McY Mcardinalis - MvY Mverbenaceus	1.319	0.613	16	2.152	0.1793
MvR Mverbenaceus - MvY Mverbenaceus	-2.638	0.613	16	-4.304	0.0028

Confidence level used: 0.95
P-value adjustment based on Tukey method for a family of four estimates.

TABLE S3. Floral morphology and nectar variation. Pairwise contrasts among floral morph/lines nested within species, based on linear mixed models (with individual plant as a random effect) followed by Type II Wald chisquare tests. Pairwise comparisons based on 95% confidence interval (CI) of least squares means following the Tukey method for p-value adjustment for multiple comparisons. Details of sample sizes in Table S1. Floral morphology measurements in mm.

Analysis of Deviance Table (Type II Wald chisquare tests)
Corolla length

	Chisq	Df	Pr(>Chisq)	
sp	10.202	1	0.001403	**
sp:line	53.997	2	1.88E-12	***

Pairwise contrasts of 95% CI of least squares means; Tukey p-value adjustment for comparing a family of 4 estimates

Contrast									
line	species		line	species	estimate	SE	df	t.ratio	p
Corolla length									
MCR	cardinalis	-	MCY	cardinalis	1.863	0.457	38.6	4.081	0.0012
MCR	cardinalis	-	MVR	verbenaceus	1.152	0.425	38.6	2.712	0.0469
MCR	cardinalis	-	MVY	verbenaceus	-1.828	0.386	16.2	-4.735	0.0011
MCY	cardinalis	-	MVR	verbenaceus	-0.711	0.554	38.9	-1.283	0.5787
MCY	cardinalis	-	MVY	verbenaceus	-3.691	0.524	23.9	-7.037	<.0001
MVR	verbenaceus	-	MVY	verbenaceus	-2.98	0.497	22.6	-5.994	<.0001

Analysis of Deviance Table (Type II Wald chisquare tests)
Display height

	Chisq	Df	Pr(>Chisq)	
sp	49.423	1	2.06E-12	***
sp:line	58.161	2	2.35E-13	***

Pairwise contrasts of 95% CI of least squares means; Tukey p-value adjustment for comparing a family of 4 estimates

Display height					estimate	SE	df	t.ratio	p
MCR	cardinalis	-	MCY	cardinalis	-2.257	0.928	29.5	-2.432	0.0928
MCR	cardinalis	-	MVR	verbenaceus	7.828	0.868	29.4	9.022	<.0001
MCR	cardinalis	-	MVY	verbenaceus	0.525	0.835	24.2	0.629	0.9217
MCY	cardinalis	-	MVR	verbenaceus	10.084	1.089	29.7	9.257	<.0001
MCY	cardinalis	-	MVY	verbenaceus	2.782	1.064	26.2	2.615	0.0655
MVR	verbenaceus	-	MVY	verbenaceus	-7.302	1.012	25.9	-7.217	<.0001

Analysis of Deviance Table (Type II Wald chisquare tests)
Display width

	Chisq	Df	Pr(>Chisq)	
sp	25.079	1	5.50E-07	***

sp:line	11.287	2	0.003541 **

Pairwise contrasts of 95% CI of least squares means; Tukey p-value adjustment for comparing a family of 4 estimates

Display width					estimate	SE	df	t.ratio	p
MCR	cardinalis	-	MCY	cardinalis	2.77	1.41	30	1.971	0.2213
MCR	cardinalis	-	MVR	verbenaceus	-1.95	1.32	30	-1.485	0.4587
MCR	cardinalis	-	MVY	verbenaceus	-6.09	1.25	22.9	-4.859	0.0004
MCY	cardinalis	-	MVR	verbenaceus	-4.73	1.65	30.3	-2.861	0.0362
MCY	cardinalis	-	MVY	verbenaceus	-8.86	1.6	25.6	-5.528	<.0001
MVR	verbenaceus	-	MVY	verbenaceus	-4.14	1.52	25.2	-2.713	0.054

Analysis of Deviance Table (Type II Wald chisquare tests)
Opening height

	Chisq	Df	Pr(>Chisq)	
sp	33.878	1	5.87E-09	***
sp:line	65.635	2	5.59E-15	***

Pairwise contrasts of 95% CI of least squares means; Tukey p-value adjustment for comparing a family of 4 estimates

Opening height					estimate	SE	df	t.ratio	p
MCR	cardinalis	-	MCY	cardinalis	-1.228	0.251	29.6	-4.887	0.0002
MCR	cardinalis	-	MVR	verbenaceus	-0.345	0.239	31.1	-1.446	0.4816
MCR	cardinalis	-	MVY	verbenaceus	-2.121	0.224	22.6	-9.476	<.0001
MCY	cardinalis	-	MVR	verbenaceus	0.883	0.298	30.8	2.958	0.0286
MCY	cardinalis	-	MVY	verbenaceus	-0.893	0.286	25.3	-3.119	0.0218
MVR	verbenaceus	-	MVY	verbenaceus	-1.776	0.276	25.8	-6.442	<.0001

Analysis of Deviance Table (Type II Wald chisquare tests)
Opening width

	Chisq	Df	Pr(>Chisq)	
sp	53.51	1	2.57E-13	***
sp:line	57.028	2	4.14E-13	***

Pairwise contrasts of 95% CI of least squares means; Tukey p-value adjustment for comparing a family of 4 estimates

Opening width					estimate	SE	df	t.ratio	p
MCR	cardinalis	-	MCY	cardinalis	-1.36	0.238	29.2	-5.707	<.0001
MCR	cardinalis	-	MVR	verbenaceus	-0.853	0.226	30.6	-3.768	0.0037
MCR	cardinalis	-	MVY	verbenaceus	-2.148	0.214	23.6	-10.033	<.0001
MCY	cardinalis	-	MVR	verbenaceus	0.508	0.283	30.3	1.797	0.2943
MCY	cardinalis	-	MVY	verbenaceus	-0.787	0.273	25.8	-2.884	0.0368
MVR	verbenaceus	-	MVY	verbenaceus	-1.295	0.262	26.3	-4.935	0.0002

Analysis of Deviance Table (Type II Wald chisquare tests)
Herkogamy

	Chisq	Df	Pr(>Chisq)

sp	2.6816	1	0.1015	
sp:line	116.3408	2	<2e-16	***

Pairwise contrasts of 95% CI of least squares means; Tukey p-value adjustment for comparing a family of 4 estimates

Herkogamy					estimate	SE	df	t.ratio	p
MCR	cardinalis	-	MCY	cardinalis	-2.281	0.419	18	-5.441	0.0002
MCR	cardinalis	-	MVR	verbenaceus	1.106	0.401	18	2.756	0.0574
MCR	cardinalis	-	MVY	verbenaceus	-2.457	0.401	18	-6.124	<.0001
MCY	cardinalis	-	MVR	verbenaceus	3.387	0.401	18	8.439	<.0001
MCY	cardinalis	-	MVY	verbenaceus	-0.177	0.401	18	-0.44	0.9707
MVR	verbenaceus	-	MVY	verbenaceus	-3.563	0.383	18	-9.313	<.0001

Analysis of Deviance Table (Type II Wald chisquare tests)
Nectar volume

	Chisq	Df	Pr(>Chisq)	
sp	48.938	1	2.64E-12	***
sp:line	2.275	2	0.3206	

Pairwise contrasts of 95% CI of least squares means; Tukey p-value adjustment for comparing a family of 4 estimates

Nectar volume (uL)					estimate	SE	df	t.ratio	p
MCR	cardinalis	-	MCY	cardinalis	4.16	3.33	24.9	1.249	0.6024
MCR	cardinalis	-	MVR	verbenaceus	16.66	3.17	27	5.251	0.0001
MCR	cardinalis	-	MVY	verbenaceus	19.95	3.33	24.9	5.987	<.0001
MCY	cardinalis	-	MVR	verbenaceus	12.5	3.9	25.7	3.205	0.0177
MCY	cardinalis	-	MVY	verbenaceus	15.78	4.03	24.4	3.917	0.0033
MVR	verbenaceus	-	MVY	verbenaceus	3.29	3.9	25.7	0.843	0.8337

Analysis of Deviance Table (Type II Wald chisquare tests)
Sucrose concentration

	Chisq	Df	Pr(>Chisq)		
sp	74.251	1	<	2.20E-16	***
sp:line	17.377	2	0.000169	***	

Pairwise contrasts of 95% CI of least squares means; Tukey p-value adjustment for comparing a family of 4 estimates

Nectar sucrose concentration					estimate	SE	df	t.ratio	p
MCR	cardinalis	-	MCY	cardinalis	0.103	0.642	24.1	0.16	0.9985
MCR	cardinalis	-	MVR	verbenaceus	5.572	0.616	27.5	9.049	<.0001
MCR	cardinalis	-	MVY	verbenaceus	2.437	0.642	24.1	3.797	0.0045
MCY	cardinalis	-	MVR	verbenaceus	5.469	0.754	25.5	7.257	<.0001
MCY	cardinalis	-	MVY	verbenaceus	2.334	0.775	23.4	3.011	0.0292
MVR	verbenaceus	-	MVY	verbenaceus	-3.135	0.754	25.5	-4.16	0.0017

TABLE S4. *Bombus* EAG responses. Pairwise contrasts among *Bombus terrestris* EAG responses to scent stimuli of all four floral morph lines (MvY, MvR, McY, McR), negative control (air), extraction solvent, and phenylacetylaldehyde (PAA, concentration 0.5 ng/uL), known to elicit a response in *Bombus terrestris ssp. audax*. All response values are corrected and scaled to PAA. Pairwise comparisons following linear mixed model, followed by Type II Wald chi-square test (see main text for details). Pairwise comparisons based on 95% confidence intervals of least squares means, using the Tukey method for p-value adjustment with Sattherwaite degrees of freedom and lmer test limit set to 6000. N= 9 bees, 1230 presentations: PAA: 315 presentations; air: 153, solvent: 153, MvY: 150, MvR: 153, McY: 153, McR: 153.

stimulus	lsmean	SE	df	lower.CL	upper.CL
air	0.694	0.103	41.6	0.487	0.901
McR	1.4	0.103	41.6	1.193	1.608
McY	1.63	0.103	41.6	1.422	1.837
MvR	1.192	0.103	41.6	0.985	1.4
MvY	1.363	0.103	41.8	1.156	1.571
PAA	0.925	0.1	37.9	0.721	1.128
solvent	0.979	0.103	41.6	0.772	1.187

contrast	estimate	SE	df	t.ratio	p.value
air-McR	-0.7062	0.127	48.4	-5.571	<.0001
air-McY	-0.9358	0.127	48.4	-7.382	<.0001
air-MvR	-0.4981	0.127	48.4	-3.929	0.0047
air-MvY	-0.6692	0.127	48.5	-5.276	0.0001
air-PAA	-0.2307	0.125	45.5	-1.848	0.5242
air-solvent	-0.2854	0.127	48.4	-2.251	0.2886
McR-McY	-0.2295	0.127	48.4	-1.811	0.5476
McR-MvR	0.2082	0.127	48.4	1.642	0.6565
McR-MvY	0.037	0.127	48.5	0.292	0.9999
McR-PAA	0.4755	0.125	45.5	3.809	0.0071
McR-solvent	0.4209	0.127	48.4	3.32	0.0267
McY-MvR	0.4377	0.127	48.4	3.453	0.0186
McY-MvY	0.2665	0.127	48.5	2.101	0.3684
McY-PAA	0.7051	0.125	45.5	5.647	<.0001
McY-solvent	0.6504	0.127	48.4	5.131	0.0001
MvR-MvY	-0.1712	0.127	48.5	-1.349	0.8251
MvR-PAA	0.2674	0.125	45.5	2.142	0.3469
MvR-solvent	0.2127	0.127	48.4	1.678	0.6336
MvY-PAA	0.4386	0.125	45.6	3.51	0.0164
MvY-solvent	0.3839	0.127	48.5	3.026	0.0565
PAA-solvent	-0.0547	0.125	45.5	-0.438	0.9994

Degrees of freedome method: sattherthwaite
Confidence level used:
0.95
P-value adjustment based on Tukey method for a family of 7 estimates.

TABLE S5. *Manduca* EAG responses. Pairwise contrasts among *Manduca sexta* EAG responses to scent stimuli of all four floral morph lines (MvY=MVYL, MvR=MVBL, McY=SM, McR=CE10), negative control (air), extraction solvent, and phenylacetylaldehyde (PAA, concentration 0.5 ng/uL), known to elicit a response in *Manduca sexta*. Pairwise comparisons based on 95% confidence intervals of least squares means, using the Tukey method for p-value adjustment with Sattherwaite degrees of freedom and lmer test limit set to 6000. Pairwise comparisons performed following linear mixed model (fixed effect: stimulus; random effects: trial and stimulus nested within trial), followed by Type II Wald chi-square test (df = 6, X^2 = 95.085, p < 2.2e-16). N = 7 moths; PAA: 189 presentations across all moths; air: 105; solvent: 105; MvY: 105; MvR: 105: McY: 102; McR: 108. Abbreviations: MvY=MVYL, MvR=MVBL, McY=SM, McR=CE10.

contrast			estimate	SE	df	t.ratio	p.value
air	-	CE10	-1.1887	0.186	36.5	-6.399	<.0001
air	-	SM	-0.8914	0.186	36.8	-4.789	0.0005
air	-	PAA	-0.2458	0.183	34.4	-1.343	0.8269
air	-	solvent	-0.1512	0.186	36.6	-0.813	0.9821
air	-	MVBL	-0.9435	0.186	36.6	-5.075	0.0002
air	-	MVYL	-0.8884	0.186	36.6	-4.778	0.0005
CE10	-	SM	0.2974	0.186	36.7	1.599	0.6838
CE10	-	PAA	0.9429	0.183	34.3	5.156	0.0002
CE10	-	solvent	1.0375	0.186	36.5	5.585	<.0001
CE10	-	MVBL	0.2453	0.186	36.5	1.32	0.8381
CE10	-	MVYL	0.3004	0.186	36.5	1.617	0.6727
SM	-	PAA	0.6456	0.183	34.6	3.523	0.0189
SM	-	solvent	0.7402	0.186	36.8	3.976	0.0053
SM	-	MVBL	-0.0521	0.186	36.8	-0.28	1
SM	-	MVYL	0.003	0.186	36.8	0.016	1
PAA	-	solvent	0.0946	0.183	34.4	0.517	0.9984
PAA	-	MVBL	-0.6977	0.183	34.4	-3.812	0.0089
PAA	-	MVYL	-0.6426	0.183	34.4	-3.511	0.0196
solvent	-	MVBL	-0.7923	0.186	36.6	-4.261	0.0024
solvent	-	MVYL	-0.7372	0.186	36.6	-3.965	0.0055
MVBL	-	MVYL	0.0551	0.186	36.6	0.296	0.9999

Degrees of freedome method: sattherthwaite
Confidence level used: 0.95
P-value adjustment based on Tukey method for a family of 7 estimates.

Table S6. **Anthocyanin biosynthesis gene expression**. Two-way ANOVA of anthocyanin biosynthesis gene expression of all four floral morphs, generated in Graphpad Prism v5.04. Swift, M. L. (1997). GraphPad prism, data analysis, and scientific graphing. Journal of chemical information and computer sciences, 37(2), 411-412.

Two-way ANOVA				
Source of Variation	% of total variation	P value		
Interaction	34.56	< 0.0001		
Column Factor	7.52	< 0.0001		
Row Factor	56.17	< 0.0001		
Source of Variation	P value summary	Significant?		
Interaction	****	Yes		
Column Factor	****	Yes		
Row Factor	****	Yes		
Source of Variation	Df	Sum-of-squares	Mean square	F
Interaction	15	1.74E+10	1.16E+09	63.11
Column Factor	3	3.78E+09	1.26E+09	68.7
Row Factor	5	2.83E+10	5.65E+09	307.7
Residual	48	8.81E+08	18360000	
Number of missing values	0			
Bonferroni multiple comparisons	Number of comparisons: 36			
MvY vs MvR				
Row Factor	MvY	MvR	Difference	95% CI of diff.

CHS	83960	30332	-53628	-65503 to -41753
CHI	1303	649.7	-653.3	-12528 to 11221
F3H	23743	11670	-12073	-23947 to -198.0
DFR	9477	5020	-4457	-16332 to 7417
ANS	7177	6487	-689.7	-12564 to 11185
UF3GT	25208	24211	-997	-12872 to 10878
Row Factor	Difference	t	P value	Summary
CHS	-53628	15.33	P < 0.0001	****
CHI	-653.3	0.1867	P > 0.05	ns
F3H	-12073	3.451	P < 0.05	*
DFR	-4457	1.274	P > 0.05	ns
ANS	-689.7	0.1971	P > 0.05	ns
UF3GT	-997	0.285	P > 0.05	ns
MvY vs McY				
Row Factor	MvY	McY	Difference	95% CI of diff.
CHS	83960	110762	26803	14928 to 38677
CHI	1303	893.7	-409.3	-12284 to 11465
F3H	23743	3002	-20741	-32616 to -8867
DFR	9477	882	-8595	-20470 to 3279
ANS	7177	4763	-2414	-14288 to 9461
UF3GT	25208	1582	-23626	-35501 to -11752
Row Factor	Difference	t	P value	Summary
CHS	26803	7.661	P < 0.0001	****
CHI	-409.3	0.117	P > 0.05	ns
F3H	-20741	5.928	P < 0.0001	****
DFR	-8595	2.457	P > 0.05	ns
ANS	-2414	0.6899	P > 0.05	ns
UF3GT	-23626	6.753	P < 0.0001	****
MvY vs McR				
Row Factor	MvY	McR	Difference	95% CI of diff.

CHS	83960	12597	-71362	-83237 to -59488
CHI	1303	893	-410	-12285 to 11465
F3H	23743	4452	-19291	-31166 to -7417
DFR	9477	2734	-6743	-18618 to 5132
ANS	7177	9849	2672	-9203 to 14547
UF3GT	25208	5646	-19562	-31437 to -7688
Row Factor	Difference	t	P value	Summary
CHS	-71362	20.4	P < 0.0001	****
CHI	-410	0.1172	P > 0.05	ns
F3H	-19291	5.514	P < 0.0001	****
DFR	-6743	1.927	P > 0.05	ns
ANS	2672	0.7637	P > 0.05	ns
UF3GT	-19562	5.591	P < 0.0001	****
MvR vs McY				
Row Factor	MvR	McY	Difference	95% CI of diff.
CHS	30332	110762	80431	68556 to 92305
CHI	649.7	893.7	244	-11631 to 12119
F3H	11670	3002	-8669	-20543 to 3206
DFR	5020	882	-4138	-16013 to 7737
ANS	6487	4763	-1724	-13599 to 10151
UF3GT	24211	1582	-22629	-34504 to -10755
Row Factor	Difference	t	P value	Summary
CHS	80431	22.99	P < 0.0001	****
CHI	244	0.06974	P > 0.05	ns
F3H	-8669	2.478	P > 0.05	ns
DFR	-4138	1.183	P > 0.05	ns
ANS	-1724	0.4928	P > 0.05	ns
UF3GT	-22629	6.468	P < 0.0001	****
MvR vs McR				
Row Factor	MvR	McR	Difference	95% CI of diff.

CHS	30332	12597	-17734	-29609 to -5860
CHI	649.7	893	243.3	-11631 to 12118
F3H	11670	4452	-7219	-19093 to 4656
DFR	5020	2734	-2286	-14160 to 9589
ANS	6487	9849	3362	-8513 to 15236
UF3GT	24211	5646	-18565	-30440 to -6691
Row Factor	Difference	t	P value	Summary
CHS	-17734	5.069	P < 0.001	***
CHI	243.3	0.06955	P > 0.05	ns
F3H	-7219	2.063	P > 0.05	ns
DFR	-2286	0.6533	P > 0.05	ns
ANS	3362	0.9609	P > 0.05	ns
UF3GT	-18565	5.307	P < 0.001	***
McY vs McR				
Row Factor	McY	McR	Difference	95% CI of diff.
CHS	110762	12597	-98165	-110040 to -86291
CHI	893.7	893	-0.6667	-11875 to 11874
F3H	3002	4452	1450	-10425 to 13325
DFR	882	2734	1852	-10022 to 13727
ANS	4763	9849	5086	-6789 to 16960
UF3GT	1582	5646	4064	-7811 to 15939
Row Factor	Difference	t	P value	Summary
CHS	-98165	28.06	P < 0.0001	****
CHI	-0.6667	0.000191	P > 0.05	ns
F3H	1450	0.4145	P > 0.05	ns
DFR	1852	0.5295	P > 0.05	ns
ANS	5086	1.454	P > 0.05	ns
UF3GT	4064	1.162	P > 0.05	ns

Table S7. **Anthocyanin biosynthesis regulators**. Two-way ANOVA of anthocyanin biosynthesis regulators of all four floral morphs, generated in Graphpad Prism v5.04. Swift, M. L. (1997). GraphPad prism, data analysis, and scientific graphing. Journal of chemical information and computer sciences, 37(2), 411-412.

Two-way ANOVA				
Source of Variation	% of total variation	P value		
Interaction	48.01	< 0.0001		
Column Factor	7.15	< 0.0001		
Row Factor	42.14	< 0.0001		
Source of Variation	P value summary	Significant?		
Interaction	****	Yes		
Column Factor	****	Yes		
Row Factor	****	Yes		
Source of Variation	Df	Sum-of-squares	Mean square	F
Interaction	18	71530000	3974000	55.3
Column Factor	3	10660000	3552000	49.43
Row Factor	6	62790000	10460000	145.6
Residual	56	4024000	71866	
Number of missing values	0			
Bonferroni multiple comparisons	Number of comparisons: 42			
MvY vs MvR				
Row Factor	MvY	MvR	Difference	95% CI of diff.
PELAN (P)	6805	2201	-4604	-5352 to -3857
PELAN (2)	7	7.667	0.6667	-747.0 to 748.3
PELAN (3)	2.333	5.667	3.333	-744.3 to 751.0
PELAN (4)	0	0.3333	0.3333	-747.3 to 748.0
PELAN (5)	0	0	0	-747.6 to

				747.6
PELAN (6)	5.667	289.7	284	-463.6 to 1032
PELAN (7)	0	0	0	-747.6 to 747.6
Row Factor	Difference	t	P value	Summary
PELAN (P)	-4604	21.04	P < 0.0001	****
PELAN (2)	0.6667	0.003046	P > 0.05	ns
PELAN (3)	3.333	0.01523	P > 0.05	ns
PELAN (4)	0.3333	0.001523	P > 0.05	ns
PELAN (5)	0	0	P > 0.05	ns
PELAN (6)	284	1.297	P > 0.05	ns
PELAN (7)	0	0	P > 0.05	ns
MvY vs McY				
Row Factor	MvY	McY	Difference	95% CI of diff.
PELAN (P)	6805	0.6667	-6805	-7552 to -6057
PELAN (2)	7	0	-7	-754.6 to 740.6
PELAN (3)	2.333	9.333	7	-740.6 to 754.6
PELAN (4)	0	0.3333	0.3333	-747.3 to 748.0
PELAN (5)	0	2.667	2.667	-745.0 to 750.3
PELAN (6)	5.667	0.6667	-5	-752.6 to 742.6
PELAN (7)	0	0	0	-747.6 to 747.6
Row Factor	Difference	t	P value	Summary
PELAN (P)	-6805	31.09	P < 0.0001	****
PELAN (2)	-7	0.03198	P > 0.05	ns
PELAN (3)	7	0.03198	P > 0.05	ns
PELAN (4)	0.3333	0.001523	P > 0.05	ns
PELAN (5)	2.667	0.01218	P > 0.05	ns
PELAN (6)	-5	0.02284	P > 0.05	ns
PELAN (7)	0	0	P > 0.05	ns
MvY vs McR				
Row Factor	MvY	McR	Difference	95% CI of diff.
PELAN (P)	6805	1048	-5758	-6505 to -5010

PELAN (2)	7	679	672	-75.64 to 1420
PELAN (3)	2.333	7.667	5.333	-742.3 to 753.0
PELAN (4)	0	4.667	4.667	-743.0 to 752.3
PELAN (5)	0	129.7	129.7	-618.0 to 877.3
PELAN (6)	5.667	0.6695	-4.997	-752.6 to 742.6
PELAN (7)	0	7.333	7.333	-740.3 to 755.0
Row Factor	Difference	t	P value	Summary
PELAN (P)	-5758	26.3	P < 0.0001	****
PELAN (2)	672	3.07	P > 0.05	ns
PELAN (3)	5.333	0.02437	P > 0.05	ns
PELAN (4)	4.667	0.02132	P > 0.05	ns
PELAN (5)	129.7	0.5924	P > 0.05	ns
PELAN (6)	-4.997	0.02283	P > 0.05	ns
PELAN (7)	7.333	0.0335	P > 0.05	ns
MvR vs McY				
Row Factor	MvR	McY	Difference	95% CI of diff.
PELAN (P)	2201	0.6667	-2200	-2948 to -1453
PELAN (2)	7.667	0	-7.667	-755.3 to 740.0
PELAN (3)	5.667	9.333	3.667	-744.0 to 751.3
PELAN (4)	0.3333	0.3333	0	-747.6 to 747.6
PELAN (5)	0	2.667	2.667	-745.0 to 750.3
PELAN (6)	289.7	0.6667	-289	-1037 to 458.6
PELAN (7)	0	0	0	-747.6 to 747.6
Row Factor	Difference	t	P value	Summary
PELAN (P)	-2200	10.05	P < 0.0001	****
PELAN (2)	-7.667	0.03503	P > 0.05	ns
PELAN (3)	3.667	0.01675	P > 0.05	ns
PELAN (4)	0	0	P > 0.05	ns
PELAN (5)	2.667	0.01218	P > 0.05	ns
PELAN (6)	-289	1.32	P > 0.05	ns
PELAN (7)	0	0	P > 0.05	ns

MvR vs McR				
Row Factor	MvR	McR	Difference	95% CI of diff.
PELAN (P)	2201	1048	-1153	-1901 to -405.7
PELAN (2)	7.667	679	671.3	-76.31 to 1419
PELAN (3)	5.667	7.667	2	-745.6 to 749.6
PELAN (4)	0.3333	4.667	4.333	-743.3 to 752.0
PELAN (5)	0	129.7	129.7	-618.0 to 877.3
PELAN (6)	289.7	0.6695	-289	-1037 to 458.6
PELAN (7)	0	7.333	7.333	-740.3 to 755.0
Row Factor	Difference	t	P value	Summary
PELAN (P)	-1153	5.269	P < 0.0001	****
PELAN (2)	671.3	3.067	P > 0.05	ns
PELAN (3)	2	0.009137	P > 0.05	ns
PELAN (4)	4.333	0.0198	P > 0.05	ns
PELAN (5)	129.7	0.5924	P > 0.05	ns
PELAN (6)	-289	1.32	P > 0.05	ns
PELAN (7)	7.333	0.0335	P > 0.05	ns
McY vs McR				
Row Factor	McY	McR	Difference	95% CI of diff.
PELAN (P)	0.6667	1048	1047	299.4 to 1795
PELAN (2)	0	679	679	-68.64 to 1427
PELAN (3)	9.333	7.667	-1.667	-749.3 to 746.0
PELAN (4)	0.3333	4.667	4.333	-743.3 to 752.0
PELAN (5)	2.667	129.7	127	-620.6 to 874.6
PELAN (6)	0.6667	0.6695	0.00282	-747.6 to 747.6
PELAN (7)	0	7.333	7.333	-740.3 to 755.0
Row Factor	Difference	t	P value	Summary
PELAN (P)	1047	4.783	P < 0.001	***
PELAN (2)	679	3.102	P > 0.05	ns
PELAN (3)	-1.667	0.007614	P > 0.05	ns

PELAN (4)	4.333	0.0198	P > 0.05	ns
PELAN (5)	127	0.5802	P > 0.05	ns
PELAN (6)	0.00282	1.29E-05	P > 0.05	ns
PELAN (7)	7.333	0.0335	P > 0.05	ns

Table S8. **Individual flavonoids**. Two-way ANOVA of individual flavonoids of all four floral morphs depicted as their retention times, generated in Graphpad Prism v5.04. Swift, M. L. (1997). GraphPad prism, data analysis, and scientific graphing. Journal of chemical information and computer sciences, 37(2), 411-412.

Two-way ANOVA				
Source of Variation	% of total variation	P value		
Interaction	47.79	< 0.0001		
Column Factor	30.53	< 0.0001		
Row Factor	17.73	< 0.0001		
Source of Variation	P value summary	Significant?		
Interaction	****	Yes		
Column Factor	****	Yes		
Row Factor	****	Yes		
Source of Variation	Df	Sum-of-squares	Mean square	F
Interaction	24	5973000	248865	72.51
Column Factor	3	3815000	1272000	370.5
Row Factor	8	2216000	277041	80.72
Residual	144	494257	3432	
Number of missing values	0			
Bonferroni multiple comparisons	Number of comparisons: 54			
MvY vs MvR				
Row Factor	MvY	MvR	Difference	95% CI of diff.
3.755	192	192.6	0.602	-124.7 to 125.9
3.995	0	0	0	-125.3 to 125.3
4.072	0	0	0	-125.3 to 125.3
4.334	0	0	0	-125.3 to 125.3
4.745	170	188.6	18.55	-106.8 to 143.9
4.85	243.9	254.4	10.48	-114.8 to 135.8
5.148	0	0	0	-125.3 to 125.3
5.774	225.2	258.5	33.32	-92.01 to 158.6
6.383	154	352.2	198.3	72.95 to 323.6

Row Factor	Difference	t	P value	Summary
3.755	0.602	0.01625	P > 0.05	ns
3.995	0	0	P > 0.05	ns
4.072	0	0	P > 0.05	ns
4.334	0	0	P > 0.05	ns
4.745	18.55	0.5006	P > 0.05	ns
4.85	10.48	0.2829	P > 0.05	ns
5.148	0	0	P > 0.05	ns
5.774	33.32	0.8992	P > 0.05	ns
6.383	198.3	5.351	P < 0.0001	****
MvY vs McY				
Row Factor	MvY	McY	Difference	95% CI of diff.
3.755	192	0	-192	-317.3 to -66.67
3.995	0	555.7	555.7	430.3 to 681.0
4.072	0	847.3	847.3	722.0 to 972.7
4.334	0	175	175	49.63 to 300.3
4.745	170	0	-170	-295.4 to -44.72
4.85	243.9	0	-243.9	-369.2 to -118.6
5.148	0	267.5	267.5	142.2 to 392.8
5.774	225.2	0	-225.2	-350.5 to -99.86
6.383	154	339.7	185.7	60.38 to 311.0
Row Factor	Difference	t	P value	Summary
3.755	-192	5.181	P < 0.0001	****
3.995	555.7	15	P < 0.0001	****
4.072	847.3	22.87	P < 0.0001	****
4.334	175	4.722	P < 0.001	***
4.745	-170	4.589	P < 0.001	***
4.85	-243.9	6.582	P < 0.0001	****
5.148	267.5	7.22	P < 0.0001	****
5.774	-225.2	6.077	P < 0.0001	****
6.383	185.7	5.012	P < 0.0001	****
MvY vs McR				
Row Factor	MvY	McR	Difference	95% CI of diff.
3.755	192	258.4	66.43	-58.90 to 191.8
3.995	0	845.3	845.3	720.0 to 970.7
4.072	0	603.1	603.1	477.7 to 728.4
4.334	0	115	115	-10.32 to

				240.3
4.745	170	222.2	52.14	-73.18 to 177.5
4.85	243.9	346	102.1	-23.24 to 227.4
5.148	0	730.7	730.7	605.4 to 856.0
5.774	225.2	306.4	81.22	-44.10 to 206.5
6.383	154	890.1	736.2	610.8 to 861.5
Row Factor	Difference	t	P value	Summary
3.755	66.43	1.793	P > 0.05	ns
3.995	845.3	22.81	P < 0.0001	****
4.072	603.1	16.28	P < 0.0001	****
4.334	115	3.104	P > 0.05	ns
4.745	52.14	1.407	P > 0.05	ns
4.85	102.1	2.755	P > 0.05	ns
5.148	730.7	19.72	P < 0.0001	****
5.774	81.22	2.192	P > 0.05	ns
6.383	736.2	19.87	P < 0.0001	****
MvR vs McY				
Row Factor	MvR	McY	Difference	95% CI of diff.
3.755	192.6	0	-192.6	-317.9 to -67.27
3.995	0	555.7	555.7	430.3 to 681.0
4.072	0	847.3	847.3	722.0 to 972.7
4.334	0	175	175	49.63 to 300.3
4.745	188.6	0	-188.6	-313.9 to -63.27
4.85	254.4	0	-254.4	-379.7 to -129.1
5.148	0	267.5	267.5	142.2 to 392.8
5.774	258.5	0	-258.5	-383.8 to -133.2
6.383	352.2	339.7	-12.57	-137.9 to 112.8
Row Factor	Difference	t	P value	Summary
3.755	-192.6	5.198	P < 0.0001	****
3.995	555.7	15	P < 0.0001	****
4.072	847.3	22.87	P < 0.0001	****
4.334	175	4.722	P < 0.001	***
4.745	-188.6	5.09	P < 0.0001	****
4.85	-254.4	6.865	P < 0.0001	****
5.148	267.5	7.22	P < 0.0001	****

5.774	-258.5	6.976	P < 0.0001	****
6.383	-12.57	0.3394	P > 0.05	ns
MvR vs McR				
Row Factor	MvR	McR	Difference	95% CI of diff.
3.755	192.6	258.4	65.82	-59.50 to 191.1
3.995	0	845.3	845.3	720.0 to 970.7
4.072	0	603.1	603.1	477.7 to 728.4
4.334	0	115	115	-10.32 to 240.3
4.745	188.6	222.2	33.6	-91.73 to 158.9
4.85	254.4	346	91.6	-33.72 to 216.9
5.148	0	730.7	730.7	605.4 to 856.0
5.774	258.5	306.4	47.9	-77.42 to 173.2
6.383	352.2	890.1	537.9	412.6 to 663.2
Row Factor	Difference	t	P value	Summary
3.755	65.82	1.776	P > 0.05	ns
3.995	845.3	22.81	P < 0.0001	****
4.072	603.1	16.28	P < 0.0001	****
4.334	115	3.104	P > 0.05	ns
4.745	33.6	0.9067	P > 0.05	ns
4.85	91.6	2.472	P > 0.05	ns
5.148	730.7	19.72	P < 0.0001	****
5.774	47.9	1.293	P > 0.05	ns
6.383	537.9	14.52	P < 0.0001	****
McY vs McR				
Row Factor	McY	McR	Difference	95% CI of diff.
3.755	0	258.4	258.4	133.1 to 383.7
3.995	555.7	845.3	289.7	164.3 to 415.0
4.072	847.3	603.1	-244.3	-369.6 to -118.9
4.334	175	115	-59.94	-185.3 to 65.38
4.745	0	222.2	222.2	96.87 to 347.5
4.85	0	346	346	220.7 to 471.3
5.148	267.5	730.7	463.2	337.8 to 588.5
5.774	0	306.4	306.4	181.1 to 431.7
6.383	339.7	890.1	550.5	425.1 to 675.8
Row Factor	Difference	t	P value	Summary

3.755	258.4	6.974	P < 0.0001	****
3.995	289.7	7.818	P < 0.0001	****
4.072	-244.3	6.592	P < 0.0001	****
4.334	-59.94	1.618	P > 0.05	ns
4.745	222.2	5.997	P < 0.0001	****
4.85	346	9.338	P < 0.0001	****
5.148	463.2	12.5	P < 0.0001	****
5.774	306.4	8.269	P < 0.0001	****
6.383	550.5	14.86	P < 0.0001	****

Table S9. **Individual carotenoids**. One-way ANOVAs of individual carotenoids of all four floral morphs, generated in Graphpad Prism v5.04. Swift, M. L. (1997). GraphPad prism, data analysis, and scientific graphing. Journal of chemical information and computer sciences, 37(2), 411-412.

Table Analyzed	Carotenoids				
Table Analyzed	Antheraxanthin RT 3.1				
One-way analysis of variance					
P value	0.0005				
P value summary	***				
Are means signif. different? (P < 0.05)	Yes				
Number of groups	4				
F	10.55				
R square	0.6642				
Bartlett's test for equal variances					
Bartlett's statistic (corrected)	5.737				
P value	0.1251				
P value summary	ns				
Do the variances differ signif. (P < 0.05)	No				
ANOVA Table	SS	df	MS		
Treatment (between columns)	951.3	3	317.1		
Residual (within columns)	480.9	16	30.06		
Total	1432	19			

Tukey's Multiple Comparison Test	Mean Diff.	q	Significant? P < 0.05?	Summary	95% CI of diff
MvR vs MvY	-13.2	5.384	Yes	**	-23.12 to -3.280
MvR vs McR	-18.82	7.676	Yes	***	-28.74 to -8.900
MvR vs McY	-12.86	5.245	Yes	**	-22.78 to -2.940
MvY vs McR	-5.62	2.292	No	ns	-15.54 to 4.300
MvY vs McY	0.34	0.1387	No	ns	-9.580 to 10.26
McR vs McY	5.96	2.431	No	ns	-3.960 to 15.88
Table Analyzed	Antheraxanthin RT 3.6				
One-way analysis of variance					
P value	< 0.0001				
P value summary	****				
Are means signif. different? (P < 0.05)	Yes				
Number of groups	4				
F	15.2				
R square	0.7402				
Bartlett's test for equal variances					
Bartlett's statistic (corrected)	2.911				
P value	0.4056				
P value summary	ns				
Do the variances differ signif. (P < 0.05)	No				

ANOVA Table	SS	df	MS		
Treatment (between columns)	371.4	3	123.8		
Residual (within columns)	130.3	16	8.146		
Total	501.8	19			
Tukey's Multiple Comparison Test	Mean Diff.	q	Significant? P < 0.05?	Summary	95% CI of diff
MvR vs MvY	-11.32	8.869	Yes	***	-16.48 to -6.156
MvR vs McR	-9.22	7.224	Yes	***	-14.38 to -4.056
MvR vs McY	-8.38	6.565	Yes	**	-13.54 to -3.216
MvY vs McR	2.1	1.645	No	ns	-3.064 to 7.264
MvY vs McY	2.94	2.303	No	ns	-2.224 to 8.104
McR vs McY	0.84	0.6581	No	ns	-4.324 to 6.004
Table Analyzed	Violaxanthin				
One-way analysis of variance					
P value	< 0.0001				
P value summary	****				
Are means signif. different? (P < 0.05)	Yes				
Number of groups	4				
F	51.45				
R square	0.9061				
Bartlett's test for equal variances					

Bartlett's statistic (corrected)	4.561				
P value	0.2069				
P value summary	ns				
Do the variances differ signif. (P < 0.05)	No				
ANOVA Table	SS	df	MS		
Treatment (between columns)	8460	3	2820		
Residual (within columns)	876.9	16	54.81		
Total	9337	19			
Tukey's Multiple Comparison Test	Mean Diff.	q	Significant? P < 0.05?	Summary	95% CI of diff
MvR vs MvY	-49.34	14.9	Yes	***	-62.74 to -35.94
MvR vs McR	-5.76	1.74	No	ns	-19.16 to 7.635
MvR vs McY	-36.12	10.91	Yes	***	-49.52 to -22.72
MvY vs McR	43.58	13.16	Yes	***	30.18 to 56.98
MvY vs McY	13.22	3.993	No	ns	-0.1754 to 26.62
McR vs McY	-30.36	9.17	Yes	***	-43.76 to -16.96
Table Analyzed	Neoxanthin				
One-way analysis of variance					
P value	0.0002				
P value summary	***				

Are means signif. different? (P < 0.05)	Yes				
Number of groups	4				
F	11.94				
R square	0.6912				
Bartlett's test for equal variances					
Bartlett's statistic (corrected)	0.6466				
P value	0.8857				
P value summary	ns				
Do the variances differ signif. (P < 0.05)	No				
ANOVA Table	SS	df	MS		
Treatment (between columns)	435.5	3	145.2		
Residual (within columns)	194.6	16	12.16		
Total	630	19			
Tukey's Multiple Comparison Test	Mean Diff.	q	Significant? P < 0.05?	Summary	95% CI of diff
MvR vs MvY	-7.32	4.694	Yes	*	-13.63 to -1.010
MvR vs McR	-2.56	1.642	No	ns	-8.870 to 3.750
MvR vs McY	-12.2	7.823	Yes	***	-18.51 to -5.890
MvY vs McR	4.76	3.052	No	ns	-1.550 to 11.07
MvY vs McY	-4.88	3.129	No	ns	-11.19 to 1.430
McR vs McY	-9.64	6.181	Yes	**	-15.95 to -3.330

Table Analyzed	Zeaxanthin				
One-way analysis of variance					
P value	< 0.0001				
P value summary	****				
Are means signif. different? (P < 0.05)	Yes				
Number of groups	4				
F	14.84				
R square	0.7357				
Bartlett's test for equal variances					
Bartlett's statistic (corrected)	8.714				
P value	0.0333				
P value summary	*				
Do the variances differ signif. (P < 0.05)	Yes				
ANOVA Table	SS	df	MS		
Treatment (between columns)	10727	3	3576		
Residual (within columns)	3854	16	240.9		
Total	14581	19			
Tukey's Multiple Comparison Test	Mean Diff.	q	Significant? P < 0.05?	Summary	95% CI of diff
MvR vs MvY	-18.56	2.674	No	ns	-46.64 to 9.522
MvR vs McR	-59.98	8.642	Yes	***	-88.06 to -31.90

MvR vs McY	-7.58	1.092	No	ns	-35.66 to 20.50
MvY vs McR	-41.42	5.968	Yes	**	-69.50 to -13.34
MvY vs McY	10.98	1.582	No	ns	-17.10 to 39.06
McR vs McY	52.4	7.55	Yes	***	24.32 to 80.48
Table Analyzed	Mimulaxanthin				
One-way analysis of variance					
P value	< 0.0001				
P value summary	****				
Are means signif. different? (P < 0.05)	Yes				
Number of groups	4				
F	79.12				
R square	0.9368				
Bartlett's test for equal variances					
Bartlett's statistic (corrected)	3.482				
P value	0.3231				
P value summary	ns				
Do the variances differ signif. (P < 0.05)	No				
ANOVA Table	SS	df	MS		
Treatment (between columns)	6142	3	2047		
Residual (within columns)	414.1	16	25.88		
Total	6556	19			

Tukey's Multiple Comparison Test	Mean Diff.	q	Significant? P < 0.05?	Summary	95% CI of diff
MvR vs MvY	-21.46	9.433	Yes	***	-30.66 to -12.26
MvR vs McR	-5	2.198	No	ns	-14.20 to 4.205
MvR vs McY	-44.9	19.74	Yes	***	-54.10 to -35.70
MvY vs McR	16.46	7.235	Yes	***	7.255 to 25.66
MvY vs McY	-23.44	10.3	Yes	***	-32.64 to -14.24
McR vs McY	-39.9	17.54	Yes	***	-49.10 to -30.70

Table S10. Carotenoid biosynthesis regulators. Two-way ANOVA of carotenoid biosynthesis regulators of all four floral morphs, generated in Graphpad Prism v5.04. Swift, M. L. (1997). GraphPad prism, data analysis, and scientific graphing. Journal of chemical information and computer sciences, 37(2), 411-412.

Two-way ANOVA				
Source of Variation	% of total variation	P value		
Interaction	17.46	< 0.0001		
Time	11.27	< 0.0001		
Row Factor	68.05	< 0.0001		
Source of Variation	P value summary	Significant?		
Interaction	****	Yes		
Time	****	Yes		
Row Factor	****	Yes		
Source of Variation	Df	Sum-of-squares	Mean square	F
Interaction	30	1831000000	61030000	15.93
Time	3	1182000000	394100000	102.9
Row Factor	10	7135000000	713500000	186.3
Residual	88	337100000	3830000	
Number of missing values	0			
Bonferroni multiple comparisons	Number of comparisons: 66			
MvY vs MvR				
Row Factor	MvY	MvR	Difference	95% CI of diff.
PSY1	2035	4474	2439	-3137 to 8016
PDS	5474	5476	1.333	-5575 to 5578
ZDS (1)	3226	1247	-1978	-7555 to 3598
ZDS (2)	2087	1526	-560.7	-6137 to 5016
ZDS (3)	10991	8313	-2679	-8255 to 2898
CrtISO	241.7	102.7	-139	-5716 to 5438

LCYB	737.3	447.7	-289.7	-5866 to 5287
BCH	17483	10905	-6578	-12155 to -1002
ZEP	34152	22178	-11974	-17551 to -6397
NSY	8851	8389	-461.9	-6039 to 5115
IspF	7546	3949	-3597	-9174 to 1979
Row Factor	Difference	t	P value	Summary
PSY1	2439	1.526	P > 0.05	ns
PDS	1.333	0.0008342	P > 0.05	ns
ZDS (1)	-1978	1.238	P > 0.05	ns
ZDS (2)	-560.7	0.3509	P > 0.05	ns
ZDS (3)	-2679	1.676	P > 0.05	ns
CrtISO	-139	0.08698	P > 0.05	ns
LCYB	-289.7	0.1813	P > 0.05	ns
BCH	-6578	4.117	P < 0.01	**
ZEP	-11974	7.493	P < 0.0001	****
NSY	-461.9	0.289	P > 0.05	ns
IspF	-3597	2.251	P > 0.05	ns
MvY vs McY				
Row Factor	MvY	McY	Difference	95% CI of diff.
PSY1	2035	9203	7168	1592 to 12745
PDS	5474	18425	12951	7374 to 18528
ZDS (1)	3226	10946	7720	2144 to 13297
ZDS (2)	2087	8460	6373	796.6 to 11950
ZDS (3)	10991	10879	-112	-5689 to 5465
CrtISO	241.7	518.7	277	-5300 to 5854
LCYB	737.3	1910	1173	-4404 to 6750
BCH	17483	38939	21455	15879 to 27032
ZEP	34152	28988	-5164	-10740 to 413.0
NSY	8851	9592	741.1	-4836 to 6318
IspF	7546	14917	7371	1794 to 12947
Row Factor	Difference	t	P value	Summary
PSY1	7168	4.486	P < 0.01	**

PDS	12951	8.104	P < 0.0001	****
ZDS (1)	7720	4.831	P < 0.001	***
ZDS (2)	6373	3.988	P < 0.01	**
ZDS (3)	-112	0.07009	P > 0.05	ns
CrtISO	277	0.1733	P > 0.05	ns
LCYB	1173	0.734	P > 0.05	ns
BCH	21455	13.43	P < 0.0001	****
ZEP	-5164	3.231	P > 0.05	ns
NSY	741.1	0.4638	P > 0.05	ns
IspF	7371	4.612	P < 0.001	***
MvY vs McR				
Row Factor	MvY	McR	Difference	95% CI of diff.
PSY1	2035	3574	1539	-4038 to 7116
PDS	5474	9015	3541	-2036 to 9118
ZDS (1)	3226	10314	7088	1511 to 12665
ZDS (2)	2087	3334	1247	-4330 to 6824
ZDS (3)	10991	6183	-4808	-10385 to 768.7
CrtISO	241.7	168	-73.67	-5650 to 5503
LCYB	737.3	858.7	121.3	-5455 to 5698
BCH	17483	17784	300.7	-5276 to 5877
ZEP	34152	14119	-20033	-25610 to -14456
NSY	8851	5636	-3214	-8791 to 2362
IspF	7546	8399	852.7	-4724 to 6429
Row Factor	Difference	t	P value	Summary
PSY1	1539	0.9631	P > 0.05	ns
PDS	3541	2.216	P > 0.05	ns
ZDS (1)	7088	4.436	P < 0.01	**
ZDS (2)	1247	0.7803	P > 0.05	ns
ZDS (3)	-4808	3.009	P > 0.05	ns
CrtISO	-73.67	0.0461	P > 0.05	ns
LCYB	121.3	0.07593	P > 0.05	ns
BCH	300.7	0.1882	P > 0.05	ns
ZEP	-20033	12.54	P < 0.0001	****
NSY	-3214	2.012	P > 0.05	ns
IspF	852.7	0.5336	P > 0.05	ns

MvR vs McY				
Row Factor	MvR	McY	Difference	95% CI of diff.
PSY1	4474	9203	4729	-847.7 to 10306
PDS	5476	18425	12950	7373 to 18526
ZDS (1)	1247	10946	9699	4122 to 15275
ZDS (2)	1526	8460	6934	1357 to 12511
ZDS (3)	8313	10879	2567	-3010 to 8143
CrtISO	102.7	518.7	416	-5161 to 5993
LCYB	447.7	1910	1463	-4114 to 7039
BCH	10905	38939	28034	22457 to 33610
ZEP	22178	28988	6810	1234 to 12387
NSY	8389	9592	1203	-4374 to 6780
IspF	3949	14917	10968	5391 to 16545
Row Factor	Difference	t	P value	Summary
PSY1	4729	2.959	P > 0.05	ns
PDS	12950	8.104	P < 0.0001	****
ZDS (1)	9699	6.069	P < 0.0001	****
ZDS (2)	6934	4.339	P < 0.01	**
ZDS (3)	2567	1.606	P > 0.05	ns
CrtISO	416	0.2603	P > 0.05	ns
LCYB	1463	0.9153	P > 0.05	ns
BCH	28034	17.54	P < 0.0001	****
ZEP	6810	4.262	P < 0.01	**
NSY	1203	0.7528	P > 0.05	ns
IspF	10968	6.864	P < 0.0001	****
MvR vs McR				
Row Factor	MvR	McR	Difference	95% CI of diff.
PSY1	4474	3574	-900.3	-6477 to 4676
PDS	5476	9015	3540	-2037 to 9116
ZDS (1)	1247	10314	9066	3490 to 14643
ZDS (2)	1526	3334	1808	-3769 to 7384
ZDS (3)	8313	6183	-2129	-7706 to

				3447
CrtISO	102.7	168	65.33	-5511 to 5642
LCYB	447.7	858.7	411	-5166 to 5988
BCH	10905	17784	6879	1302 to 12456
ZEP	22178	14119	-8059	-13636 to -2482
NSY	8389	5636	-2753	-8329 to 2824
IspF	3949	8399	4450	-1127 to 10027
Row Factor	Difference	t	P value	Summary
PSY1	-900.3	0.5634	P > 0.05	ns
PDS	3540	2.215	P > 0.05	ns
ZDS (1)	9066	5.674	P < 0.0001	****
ZDS (2)	1808	1.131	P > 0.05	ns
ZDS (3)	-2129	1.332	P > 0.05	ns
CrtISO	65.33	0.04088	P > 0.05	ns
LCYB	411	0.2572	P > 0.05	ns
BCH	6879	4.305	P < 0.01	**
ZEP	-8059	5.043	P < 0.001	***
NSY	-2753	1.723	P > 0.05	ns
IspF	4450	2.785	P > 0.05	ns
McY vs McR				
Row Factor	McY	McR	Difference	95% CI of diff.
PSY1	9203	3574	-5629	-11206 to -52.65
PDS	18425	9015	-9410	-14987 to -3833
ZDS (1)	10946	10314	-632.3	-6209 to 4944
ZDS (2)	8460	3334	-5126	-10703 to 450.4
ZDS (3)	10879	6183	-4696	-10273 to 880.7
CrtISO	518.7	168	-350.7	-5927 to 5226
LCYB	1910	858.7	-1052	-6628 to 4525
BCH	38939	17784	-21155	-26731 to -15578
ZEP	28988	14119	-14869	-20446 to -9293
NSY	9592	5636	-3956	-9532 to 1621
IspF	14917	8399	-6518	-12095 to -941.3

Row Factor	Difference	t	P value	Summary
PSY1	-5629	3.523	P < 0.05	*
PDS	-9410	5.889	P < 0.0001	****
ZDS (1)	-632.3	0.3957	P > 0.05	ns
ZDS (2)	-5126	3.208	P > 0.05	ns
ZDS (3)	-4696	2.939	P > 0.05	ns
CrtISO	-350.7	0.2194	P > 0.05	ns
LCYB	-1052	0.6581	P > 0.05	ns
BCH	-21155	13.24	P < 0.0001	****
ZEP	-14869	9.305	P < 0.0001	****
NSY	-3956	2.475	P > 0.05	ns
IspF	-6518	4.079	P < 0.01	**

Supplemental Data 11. **Statistical Analyses of genomic variants by 50kb window, per chromosome.** Generated in Graphpad Prism v5.04. Swift, M. L. (1997). GraphPad prism, data analysis, and scientific graphing. Journal of chemical information and computer sciences, 37(2), 411-412. See separate SupplementalData11.xlsx file

Supplemental Data 12. ITS Fasta sequences used to generate Maximum Likelihood Tree (Figure S1B). See separate SupplementalData12.txt file.

Table S13. Full floral volatile compound list for *Mimulus verbenaceus* (expanded version of Table 2). Numbers in parentheses after each emission value indicate the number of samples a compound was found in from that line (total sample numbers for each line are in the table header). Under the Compound Name header, superscript letters: A: compound identity validated using authentic reference standards; B: compound identity validated using published Kovats Retention Indices, our calculated Kovats Retention Indices, and NIST Library spectrum matching; C: compound identity could not be validated and compound is listed as an unknown . Values in bold text differed significantly between red and yellow morphs. Note for Cymene: m-/o-/p- structure could not be differentiated with standards. MT: monoterpenoid; Unk: unknown.

**Compound Name**	**Type**	**Kovats Retention Index**	**Yellow *M. verbenaceus* MvY (n = 17)**	**Red *M. verbenaceus* MvR (n = 18)**
Total emissions (summed across all compounds)	-	-	**675.830 ± 43.538**	**291.169 ± 19.487**
Cymene^A (m-/o-/p- structure could not be determined with standards)	aromatic	1021	**7.096±0.860 (16)**	**2.374±0.361 (17)**
Cuminaldehyde^A	aromatic	1236	**1.784±0.280 (15)**	**0.407±0.125 (10)**
Cuminol^A	aromatic	1290	**1.037±0.211 (13)**	**0.071±0.042 (3)**
1-Octen-3-ol^A	FAD	979	**3.342±0.397 (16)**	**0.299±0.180 (3)**
Tridecane^A	FAD	1300	**0.496±0.144 (10)**	**1.699±0.413 (12)**
α-Thujene^A	terpenoid	923	**55.323±5.055 (17)**	**12.770±2.420 (18)**
α-Pinene^A	terpenoid	929	**254.941±18.331 (17)**	**126.776±8.250 (18)**
Camphene^A	terpenoid	942	**2.132±0.288 (16)**	**0.580±0.044 (18)**
Sabinene^A	terpenoid	971	**114.602±9.888 (17)**	**51.895±4.510 (18)**
β-Pinene^A	terpenoid	972	**26.593±3.216 (17)**	**17.861±0.767 (18)**
β-Myrcene^A	terpenoid	990	**3.630±0.267 (17)**	**1.365±0.176 (15)**
α-Terpinene^A	terpenoid	1013	**1.670±0.278 (13)**	**0.395±0.114 (11)**
β-Phellandrene^A	terpenoid	1025	**52.585±3.663 (17)**	**11.589±1.592 (18)**
Eucalyptol^A	terpenoid	1026	**2.797±0.304 (17)**	**0.750±0.089 (18)**

γ-Terpinene[A]	terpenoid	1055	**2.050±0.405 (12)**	**0.125±0.071 (3)**
Sabinene hydrate[A]	terpenoid	1063	**34.796±5.026 (15)**	**8.154±1.800 (11)**
Terpinolene[A]	terpenoid	1085	**0.587±0.133 (13)**	**0.068±0.032 (4)**
Pinene oxide[A]	terpenoid	1092	**12.466±1.461 (17)**	**5.428±0.545 (17)**
Unknown monoterpenoid[C]	terpenoid	1095	**9.281±1.130 (17)**	**2.636±0.337 (17)**
β-Thujone[A]	terpenoid	1113	**1.146±0.190 (15)**	**0.311±0.067 (14)**
α-Campholenal[B]	terpenoid	1123	**5.080±0.513 (17)**	**1.864±0.267 (18)**
Nopinone[A]	terpenoid	1133	7.468±0.526 (17)	5.563±1.046 (18)
Sabinol[B]	terpenoid	1137	**2.111±0.397 (15)**	**Absent (0)**
(*E*)-Verbenol[A]	terpenoid	1142	**2.493±0.221 (17)**	**0.175±0.101 (3)**
Sabine ketone[B]	terpenoid	1153	21.484±2.918 (17)	14.803±2.303 (18)
Pinocarvone[B]	terpenoid	1157	**2.041±0.178 (17)**	**0.555±0.128 (12)**
Myrtenal isomer 1[A]	terpenoid	1181	**1.105±0.178 (14)**	**0.188±0.109 (3)**
Cryptone[B]	terpenoid	1182	**3.186±0.381 (17)**	**1.597±0.179 (18)**
α-Terpineol[A]	terpenoid	1188	**0.070±0.010 (16)**	**Absent (0)**
Myrtenal isomer 2[A]	terpenoid	1191	**5.490±0.436 (17)**	**1.115±0.402 (6)**
Verbenone[A]	terpenoid	1205	**4.426±0.784 (12)**	**0.268±0.184 (2)**
M. verbenaceus Unknown 1[C]	unknown	894	**1.047±0.219 (14)**	**0.139±0.045 (7)**
M. verbenaceus Unknown 2[C]	unknown	1176	**1.130±0.168 (15)**	**0.438±0.126 (9)**
M. verbenaceus Unknown 3[C]	unknown	1273	3.348±0.448 (16)	3.922±0.983 (15)
M. verbenaceus Unknown 4[C]	unknown	1312	1.710±0.228 (15)	1.375±0.173 (18)
M. verbenaceus Unknown 5[C]	unknown	1392	**23.187±3.656 (16)**	**0.521±0.357 (2)**
M. verbenaceus Unknown 6[C]	unknown	1396	1.517±0.460 (11)	1.256±0.212 (14)
M. verbenaceus	unknown	1942	**0.537±0.207 (8)**	**9.071±1.868 (17)**

Unknown 7[C]				
M. verbenaceus Unknown 8[C]	unknown	2028	**0.044±0.030 (2)**	**2.766±0.607 (16)**
