# Supplementary material for: Within-species floral evolution reveals convergence in adaptive walks during incipient pollinator shift
Source: Nat Commun. 2025 Mar 19;16:2721. doi: 10.1038/s41467-025-57639-3 (PMC11923230; doi:10.1038/s41467-025-57639-3)
Supplement: Supplementary file 2 — Description of Additional Supplementary Files [file 41467_2025_57639_MOESM2_ESM.pdf]

Description of Additional Supplementary Files

File name: Supplementary Data 1
Description: Statistical Analyses of genomic variants by 50kb window, per chromosome. Generated in Graphpad Prism v5.04. Swift, M. L. (1997). GraphPad prism, data analysis, and scientific graphing. Journal of chemical information and computer sciences, 37(2), 411-412.

File Name: Supplementary Data 2
Description: ITS Fasta sequences used to generate Maximum Likelihood Tree (Figure S1B).
