# Supplementary material for: Within-species floral evolution reveals convergence in adaptive walks during incipient pollinator shift
Source: Nat Commun. 2025 Mar 19;16:2721. doi: 10.1038/s41467-025-57639-3 (PMC11923230; doi:10.1038/s41467-025-57639-3)
Supplement: Supplementary file 5 — Reporting Summary [file 41467_2025_57639_MOESM5_ESM.pdf]

nature portfolio

Corresponding author(s): Kelsey J.R.P. Byers

Last updated by author(s): Jan 28, 2025

Reporting Summary

Nature Portfolio wishes to improve the reproducibility of the work that we publish. This form provides structure for consistency and transparency in reporting. For further information on Nature Portfolio policies, see our Editorial Policies and the Editorial Policy Checklist.

Statistics

For all statistical analyses, confirm that the following items are present in the figure legend, table legend, main text, or Methods section.

n/a	Confirmed	
[ ]	[x]	The exact sample size (*n*) for each experimental group/condition, given as a discrete number and unit of measurement
[ ]	[x]	A statement on whether measurements were taken from distinct samples or whether the same sample was measured repeatedly
[ ]	[x]	The statistical test(s) used AND whether they are one- or two-sided<br>*Only common tests should be described solely by name; describe more complex techniques in the Methods section.*
[ ]	[x]	A description of all covariates tested
[ ]	[x]	A description of any assumptions or corrections, such as tests of normality and adjustment for multiple comparisons
[ ]	[x]	A full description of the statistical parameters including central tendency (e.g. means) or other basic estimates (e.g. regression coefficient) AND variation (e.g. standard deviation) or associated estimates of uncertainty (e.g. confidence intervals)
[ ]	[x]	For null hypothesis testing, the test statistic (e.g. *F*, *t*, *r*) with confidence intervals, effect sizes, degrees of freedom and *P* value noted<br>*Give P values as exact values whenever suitable.*
[x]	[ ]	For Bayesian analysis, information on the choice of priors and Markov chain Monte Carlo settings
[x]	[ ]	For hierarchical and complex designs, identification of the appropriate level for tests and full reporting of outcomes
[x]	[ ]	Estimates of effect sizes (e.g. Cohen's *d*, Pearson's *r*), indicating how they were calculated

Our web collection on statistics for biologists contains articles on many of the points above.

Software and code

Policy information about availability of computer code

Data collection	Reflectance spectrophotometry data were collected using OceanView 2.0.8 software. Electroantennographic data were collected using GcEAD 2014 1.2.5 software.
Data analysis	Data were analysed in R 4.0.5 using the psych 2.2.5 car 3.0.10 pavo 2.7.1 lme4 1.1-27.1 lmerTest 0.9-38 and vegan 2.5-7 packages. kallisto 0.46.1 was used to analyse RNAseq data and degust 4.1.1 (web application) was used to visualise RNAseq data. bwa 0.7.17, samtools 1.7, bcftools 1.10.2, bedtools 2.27.1, Mafft 7.250, fasttree 2.1.9, and Interactive Tree of Life (iTOL) 6.9 (web application) were used to analyse and visualise WGS data. Agilent Unknowns software 10.1 and the NIST 2017 library were used for analysing GCMS data (with transformations into Excel done using a custom Perl script with no data analysis role that is now available on Zenodo). Masshunter Workstation Qualitative Analysis 10.0 was used to analyse HPLC data.

For manuscripts utilizing custom algorithms or software that are central to the research but not yet described in published literature, software must be made available to editors and reviewers. We strongly encourage code deposition in a community repository (e.g. GitHub). See the Nature Portfolio guidelines for submitting code & software for further information.

Data

Policy information about availability of data

All manuscripts must include a data availability statement. This statement should provide the following information, where applicable:

- Accession codes, unique identifiers, or web links for publicly available datasets
- A description of any restrictions on data availability
- For clinical datasets or third party data, please ensure that the statement adheres to our policy

Data generated during the course of the research discussed in this manuscript are archived publicly. Sequence data are archived with the European Nucleotide Archive (ENA, accession PRJEB75514, https://www.ebi.ac.uk/ena/browser/view/PRJEB75514). Metabolomic data and reflectance spectrophotometry have been deposited with figshare (UHPLC-MS data: https://doi.org/10.6084/m9.figshare.28287782.v1, GC-MS data: https://doi.org/10.6084/m9.figshare.28287506.v1, and reflectance spectrophotometry data: https://doi.org/10.6084/m9.figshare.28287470.v1) and source data (excluding sequencing and metabolomic data) are additionally provided provided in the Supplementary Information/Source Data file with this paper.

Research involving human participants, their data, or biological material

Policy information about studies with human participants or human data. See also policy information about sex, gender (identity/presentation), and sexual orientation and race, ethnicity and racism.

Reporting on sex and gender

Use the terms sex (biological attribute) and gender (shaped by social and cultural circumstances) carefully in order to avoid confusing both terms. Indicate if findings apply to only one sex or gender; describe whether sex and gender were considered in study design; whether sex and/or gender was determined based on self-reporting or assigned and methods used.

Provide in the source data disaggregated sex and gender data, where this information has been collected, and if consent has been obtained for sharing of individual-level data; provide overall numbers in this Reporting Summary. Please state if this information has not been collected.

Report sex- and gender-based analyses where performed, justify reasons for lack of sex- and gender-based analysis.

Reporting on race, ethnicity, or other socially relevant groupings

Please specify the socially constructed or socially relevant categorization variable(s) used in your manuscript and explain why they were used. Please note that such variables should not be used as proxies for other socially constructed/relevant variables (for example, race or ethnicity should not be used as a proxy for socioeconomic status).

Provide clear definitions of the relevant terms used, how they were provided (by the participants/respondents, the researchers, or third parties), and the method(s) used to classify people into the different categories (e.g. self-report, census or administrative data, social media data, etc.)

Please provide details about how you controlled for confounding variables in your analyses.

Population characteristics

Describe the covariate-relevant population characteristics of the human research participants (e.g. age, genotypic information, past and current diagnosis and treatment categories). If you filled out the behavioural & social sciences study design questions and have nothing to add here, write "See above."

Recruitment

Describe how participants were recruited. Outline any potential self-selection bias or other biases that may be present and how these are likely to impact results.

Ethics oversight

Identify the organization(s) that approved the study protocol.

Note that full information on the approval of the study protocol must also be provided in the manuscript.

Field-specific reporting

Please select the one below that is the best fit for your research. If you are not sure, read the appropriate sections before making your selection.

☐ Life sciences ☐ Behavioural & social sciences ☒ Ecological, evolutionary & environmental sciences

For a reference copy of the document with all sections, see nature.com/documents/nr-reporting-summary-flat.pdf

Ecological, evolutionary & environmental sciences study design

All studies must disclose on these points even when the disclosure is negative.

Study description

Floral phenotype data (morphology, nectar, scent, pigments, reflectance) were collected from 5-15 individuals per line (see below for description of samples). In addition, electroantennography was done using scent samples presented to Bombus terrestris audax bumble bees and Manduca sexta hawkmoths. Behaviour experiments were done using Bombus terrestris audax bumble bees choosing within species between lines of each species. Finally, whole genome sequencing was combined with existing genomes (CE10, MVBL) and whole transcriptome sequencing was done de novo for three replicates of each line.

Research sample

Samples were taken from four inbred lines of Mimulus section Erythranthe grown in glasshouse conditions: MVYL (yellow Mimulus verbenaceus, representing the single yellow population known of this species); MVBL (red Mimulus verbenaceus, representing the dominant phenotype of this species); SM (yellow Mimulus cardinalis, representing one of two yellow populations known of this species); and CE10 (red Mimulus cardinalis, representing the dominant phenotype of this species). Lines were inbred for at least 10

generations prior to growth and sampling. Publicly available genomes for CE10 and MVBL were used (downloaded from www.mimubase.org).

Sampling strategy	Data were collected from 5-15 plants per line and in triplicate from each plant, with sample sizes as in Table S1. Sample sizes were typical for the experiments conducted, including behaviour and electroantennography as well as transcriptomics (3 samples per line), and in line with what is typical for the literature in these areas.
Data collection	Data were collected from glasshouse-grown plants of the four lines by authors KEW, MN, and KJRPB using methods described fully in the manuscript. All data were collected from excised (cut) flowers rather than from intact (on-plant) flowers.
Timing and spatial scale	Data were collected from first-day-open flowers (i.e. the first day of anthesis) from plants grown at various times of year in controlled glasshouse conditions.
Data exclusions	The first three puffs of each electroantennography scent delivery were excluded as they represent solvent blow-off and not the insect's response to the actual sample (see Byers, Darragh et al. 2020 Evolution). No other data were excluded.
Reproducibility	Experiments were not reproduced, but appropriate sample sizes were used to ensure internal consistency.
Randomization	Randomization for electroantennography (stimulus presentation order) and behavioural experiments was done using a custom Perl script utilizing the drand48 algorithm which is a linear congruential algorithm.
Blinding	Behavioural observation, sample collection, and data analysis were not blinded as the flowers are visually quite distinct so blinding was not possible.

Did the study involve field work? ☐ Yes ☒ No

Reporting for specific materials, systems and methods

We require information from authors about some types of materials, experimental systems and methods used in many studies. Here, indicate whether each material, system or method listed is relevant to your study. If you are not sure if a list item applies to your research, read the appropriate section before selecting a response.

Materials & experimental systems

n/a	Involved in the study	
☒	☐	Antibodies
☒	☐	Eukaryotic cell lines
☒	☐	Palaeontology and archaeology
☐	☒	Animals and other organisms
☒	☐	Clinical data
☒	☐	Dual use research of concern
☐	☒	Plants

Methods

n/a	Involved in the study	
☒	☐	ChIP-seq
☒	☐	Flow cytometry
☒	☐	MRI-based neuroimaging

Animals and other research organisms

Policy information about studies involving animals; ARRIVE guidelines recommended for reporting animal research, and Sex and Gender in Research

Laboratory animals	Bombus terrestris audax workers were used for behavioural and electroannenographic studies. Manduca sexta were used for electroantennographic studies.
Wild animals	none used
Reporting on sex	Behavioural and electroatennographic studies used only female worker bumble bees as these are the caste that collects pollen and nectar while males collect only nectar. Female Manduca sexta were used for electroantennographic experiments as they are less likely to contain biased olfactory receptor repertoires since they are not also seeking to detect female moth pheromones.
Field-collected samples	none used
Ethics oversight	No ethical oversight was required as these are invertebrate animals (insects); however, they were handled in an appropriate and respectful fashion and euthanised by freezing and only minimum numbers needed were used.

Note that full information on the approval of the study protocol must also be provided in the manuscript.

Dual use research of concern

Policy information about dual use research of concern

Hazards

Could the accidental, deliberate or reckless misuse of agents or technologies generated in the work, or the application of information presented in the manuscript, pose a threat to:

No	Yes	
☒	☐	Public health
☒	☐	National security
☒	☐	Crops and/or livestock
☒	☐	Ecosystems
☒	☐	Any other significant area

Experiments of concern

Does the work involve any of these experiments of concern:

No	Yes	
☒	☐	Demonstrate how to render a vaccine ineffective
☒	☐	Confer resistance to therapeutically useful antibiotics or antiviral agents
☒	☐	Enhance the virulence of a pathogen or render a nonpathogen virulent
☒	☐	Increase transmissibility of a pathogen
☒	☐	Alter the host range of a pathogen
☒	☐	Enable evasion of diagnostic/detection modalities
☒	☐	Enable the weaponization of a biological agent or toxin
☒	☐	Any other potentially harmful combination of experiments and agents

Plants

Seed stocks: The yellow-flowered color morph of M. verbenaceus (MVYL, hereafter MvY) was originally collected from a population located at Vassey's Paradise in the Grand Canyon, Arizona, USA (exact coordinates unknown), near the northwestern extent of the species range (Vickery and Vickery, 1992), about 100 miles north of the source population of the red-flowered M. verbenaceus (MVBL, hereafter MvR, Figure S1A). MvR was collected along the West Fork Trail in Sedona, AZ, USA (34.9883°N, 111.7485°W). Yellow-flowered morphs of M. cardinalis have been characterized at two populations: one at the northern extent of the species range in the Siskiyou Mountains of Jackson County, Oregon, USA (known as SM, hereafter McY), and one from the southern range extent in Cedros Island, Baja California. For this study, we characterize the yellow morph from the northern population (SM, exact coordinates unknown), though we note that previous work with pollinators include the southern Cedros Island population (Vickery and Vickery, 1992; Vickery, 1995). The red-flowered reference line of M. cardinalis (CE10, hereafter McR) was collected near South Fork Tuolumne River, Tuolumne County, California, USA (37.817°N, 119.867°W).

Novel plant genotypes: no novel plant genotypes were generated.

Authentication: Seeds were not authenticated; however, Byers has seen these lines growing at other institutions (University of Washington) and they are visually identical to reference photographs taken by Byers previously. Seeds were provided by collaborators with extensive experience working with these lines.
